# Glutamate-Bound NMDARs Arising from *In Vivo*-like Network Activity Extend Spatio-temporal Integration in a L5 Cortical Pyramidal Cell Model

**DOI:** 10.1371/journal.pcbi.1003590

**Published:** 2014-04-24

**Authors:** Matteo Farinella, Daniel T. Ruedt, Padraig Gleeson, Frederic Lanore, R. Angus Silver

**Affiliations:** Department of Neuroscience, Physiology and Pharmacology, University College London, London, United Kingdom; Université Paris Descartes, Centre National de la Recherche Scientifique, France

## Abstract

*In vivo*, cortical pyramidal cells are bombarded by asynchronous synaptic input arising from ongoing network activity. However, little is known about how such ‘background’ synaptic input interacts with nonlinear dendritic mechanisms. We have modified an existing model of a layer 5 (L5) pyramidal cell to explore how dendritic integration in the apical dendritic tuft could be altered by the levels of network activity observed *in vivo*. Here we show that asynchronous background excitatory input increases neuronal gain and extends both temporal and spatial integration of stimulus-evoked synaptic input onto the dendritic tuft. Addition of fast and slow inhibitory synaptic conductances, with properties similar to those from dendritic targeting interneurons, that provided a ‘balanced’ background configuration, partially counteracted these effects, suggesting that inhibition can tune spatio-temporal integration in the tuft. Excitatory background input lowered the threshold for NMDA receptor-mediated dendritic spikes, extended their duration and increased the probability of additional regenerative events occurring in neighbouring branches. These effects were also observed in a passive model where all the non-synaptic voltage-gated conductances were removed. Our results show that glutamate-bound NMDA receptors arising from ongoing network activity can provide a powerful spatially distributed nonlinear dendritic conductance. This may enable L5 pyramidal cells to change their integrative properties as a function of local network activity, potentially allowing both clustered and spatially distributed synaptic inputs to be integrated over extended timescales.

## Author Summary

In the brains of awake animals, networks are active even when there is no input from the outside world. Neurons embedded within cortical networks experience this intrinsic ongoing firing as ‘background’ synaptic input. While the effect of this background input on the integration properties of neurons has been studied in the cell body region, little is known about how asynchronous background activity affects integration in distal dendrites, which contain nonlinear mechanisms that boost and dampen synaptic input. Our simulations, using a model of a cortical L5 pyramidal cell, show that the nonlinear NMDA receptor conductance activated by distributed background activity could increase the gain of the dendrite, enabling synaptic inputs to be integrated more effectively over the dendritic tree and over longer time intervals than previously thought possible. This mechanism could potentially enable the integrative properties of individual neurons to change as a function of the activity of the network in which they are embedded. Our work suggests that background network activity could play a key role routing and transforming information as it flows through the cortex.

## Introduction

Pyramidal cells are the principal excitatory neurons in the cerebral cortex and those in layer 5 (L5) form its primary output [1]–[3]. Tufted L5 pyramidal cells integrate synaptic input from local circuits together with long-range inputs from other cortical regions and thalamic nuclei [4], [5]. A substantial fraction of the long range thalamic and cortico-cortical inputs form synapses onto the highly branched apical tuft in L1 and L2 [6], [7], which is electrically remote from the soma [8]. The tuft therefore receives many different types of signals including information on different sensory modalities, motor control and emotional state [5], [9], [10]. These inputs are likely to span a wide range of temporal scales, ranging from precisely timed millisecond bursts to more sustained rate-coded signals. This raises the question as to how an individual tufted L5 pyramidal cell combines and transforms such temporally and spatially diverse signals.

*In vivo*, cortical neurons are constantly bombarded with synaptic activity [11]–[13]. This ‘background’ synaptic input reflects both the intrinsic network activity of the thalamocortical system [14] and extrinsic drive. Background input in cortex typically consists of both excitatory and inhibitory synaptic input in an approximately balanced configuration [15]–[18]. The shunt and voltage noise introduced by the synaptic membrane conductances [19], [20] alter the electrotonic properties of the cell [21], [22] and can change the arithmetic operations that a neuron can perform [20], [23]–[28]. However, little is known about how background synaptic input affects nonlinear dendritic mechanisms in fine dendrites [28].

*In vitro* studies show that voltage-dependent synaptic NMDA receptors (NMDARs) can sustain local regenerative responses called NMDAR spikes in the fine dendrites of pyramidal cells [29]–[33]. Such dendritic thresholding units could substantially increase the computational power of the neuron by enabling the dendritic tree to act like a feed-forward neural network [33], [34]. Dendritic recordings and two-photon uncaging experiments in acute slices indicate that activation of NMDAR spikes requires input from a substantial number of clustered synapses [29], [31], [35], [36] and that activation of multiple branches are required to trigger a Ca^2+^ spike in the apical dendrite [29], [37], which couples the electrically remote tuft to the axon initial segment, where action potentials (APs) are generated [38]. On the other hand, *in vivo* experiments show that synaptic input evoked by sensory stimuli are dispersed over the dendritic tree of pyramidal cells in visual [39] and barrel cortices [40] as well as spontaneous synaptic input in L5 neurons of motor cortex [41], casting doubt on whether such highly correlated spatio-temporal patterns of synaptic input occur naturally in cortex, although they have been reported in hippocampus [42]. Moreover, NMDAR spikes are also sensitive to inhibition [43], [44] and are therefore likely to be less robust *in vivo*, where inhibition is stronger [16], [17], than under *in vitro* conditions where inhibition is often reduced or absent. Nevertheless, NMDAR spikes have been reported in L4 spiny stellate neurons of barrel cortex following whisker stimulation [45] and in L2/3 pyramidal cells in somatosensory cortex during hind-limb stimulation [46]. Moreover, dendritic patch-clamp recordings from L2/3 pyramidal cells in primary visual cortex suggest that NMDAR spikes contribute to orientation selectivity [47]. Large and widespread Ca^2+^ transients have been recorded in the tufts of L5 pyramidal cells when sensory input occurs during motor activity, but evidence suggests that Ca^2+^ spikes rather than NMDAR spikes were responsible [10]. Indeed, inhibition of dendritic targeting somatostatin expressing (SOM) interneurons, which have been shown to transiently reduce inhibitory tone in the dendrites of L2/3 cells during active behaviour [48], could underlie the increase in gain of the tuft region of L5 pyramidal cells. These findings show that NMDAR spikes occur in a range of different cortical neurons *in vivo*, but it is unclear to what extent nonlinear NMDAR conductances contribute to synaptic integration in L5 pyramidal cells and what spatio-temporal patterns these cells respond to *in vivo*.

We have investigated the potential impact of *in vivo*-like background network activity on synaptic integration in L5 cortical pyramidal cells using a morphologically and biophysically detailed model that reproduces a wide range of experimentally measured synaptic, dendritic and somatic behaviours [29]. Our simulations show that asynchronous background synaptic input onto the apical dendrites at rates observed *in vivo* [48]–[50] can profoundly alter the integrative properties of L5 pyramidal cells. Our results suggest that, by activating nonlinear NMDAR conductances distributed over the dendritic tuft, background excitatory input enables pyramidal cells to integrate spatio-temporally distributed patterns of synaptic input in L1 and L2/3 and that the level of background inhibition could tune the spatio-temporal integration window.

## Results

**Figure 1:**
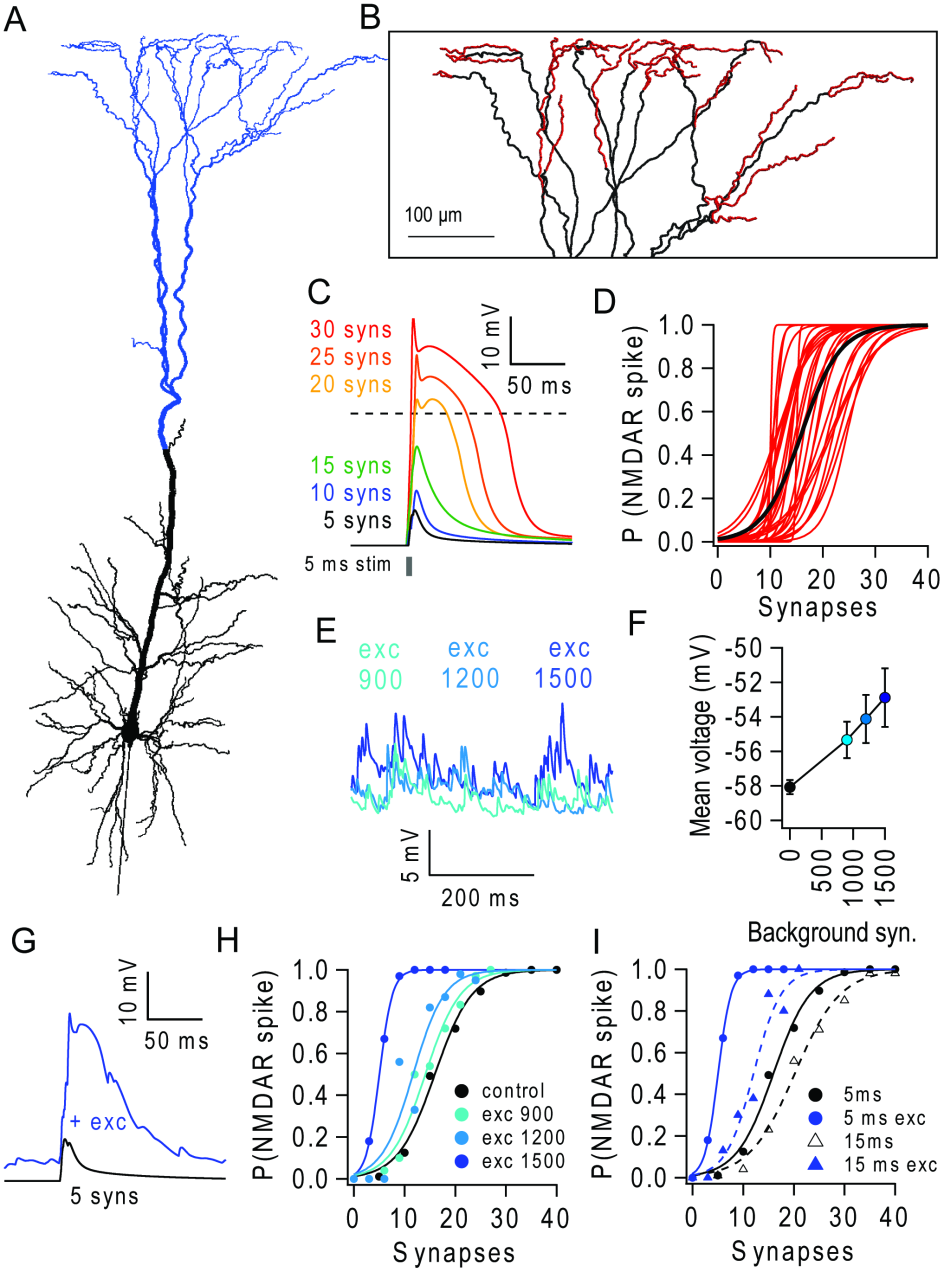
Background excitatory input lowers NMDAR spike threshold and increases gain of the input-output relationship of apical dendrites. (**A**) Morphology of L5 pyramidal neuron model [29] with apical tuft highlighted in blue. (**B**) The 28 terminal branches (red) on the dendritic tuft. (**C**) Membrane potential in an apical branch for different numbers of near-synchronous stimulated (5 ms window) quantal AMPAR/NMDAR synaptic inputs. Dashed line shows NMDAR spike threshold criterion (−30 mV). (**D**) Probability of NMDAR spike (P(NMDAR spike)) versus number of stimulated synapses randomly distributed along the branch. Red lines show fits for each of the 28 branches. Black line denotes average across all branches. (**E**) Membrane potential from a single branch during different levels of background excitatory input (exc, 900, 1200 and 1500 synapses firing at 0.85 Hz) distributed on the apical tuft (blue region in A). (**F**) Mean and standard deviation of branch voltage for different levels of background activity. (**G**) EPSP during control (black) and NMDAR spike during background excitatory input (blue) on the apical tuft. (**H**) Mean branch I-O relationship for 5 ms window (computed from ∼50 trials across randomly selected branches) during different levels of background input (as indicated). (**I**) Same as H but comparing results for a 5 ms stimulation window (filled circles, solid lines) and 15 ms window (open circles, dashed lines) for 1500 background inputs.

To investigate how background network activity influences synaptic integration in tufted L5 cortical pyramidal cells, we adapted an existing model consisting of a detailed morphology (**Fig. 1A**) and 9 membrane conductances ([29] **Text S1. Supporting Information**). Groups of synaptic inputs were generated with the software neuroConstruct [51] to mimic stimulus-evoked L1/2 input (i.e. cortico-cortico or thalamo-cortical sensory, motor or emotional) and ongoing background network activity onto the apical dendritic tree. Excitatory synaptic inputs were simulated with random Poisson trains of postsynaptic conductances, consisting of a linear AMPA receptor (AMPAR) component and a voltage-dependent NMDAR component, with peak amplitudes and kinetics that matched experimentally measured quantal excitatory postsynaptic current (EPSC) waveforms and the AMPAR/NMDAR ratio in these cells [29], [35], [52]–[54]. Single quantal conductances were used because the ∼5 synaptic contacts that typically make up a unitary synaptic connection between two cortical pyramidal cells are usually distributed over different dendritic branches [53]. We first compared integration of excitatory synaptic input in the tuft under quiescent conditions and during modest levels of background excitatory synaptic input, to enable comparison to *in vitro* experimental results. However, to understand the role of background input under more physiological conditions, we then examined the properties of pyramidal cell integration when background excitation and inhibition were balanced, as observed *in vivo*.

### Number of synapses required to trigger NMDAR spikes in distal dendritic branches

We examined how many synapses onto an individual terminal dendritic branch in the apical tuft were required to trigger a regenerative NMDAR event in the absence of background synaptic input. This condition roughly approximates the *in vitro* conditions used to study synaptic integration with glutamate uncaging, where background activity is reduced due to long range inputs being severed in acute slices and inhibition is blocked by MNI-glutamate [55]. Since the most likely location for NMDAR spike generation is in the high impendence terminal branches of the tuft [29], [35], [56], we focused on these dendritic compartments (**Fig. 1B**). To stimulate a particular branch, synapses were randomly distributed along the branch and near-coincident stimulus-evoked synaptic input was mimicked by driving each synapse with a 200 Hz random train for 5 ms. This resulted in each quantal synaptic input (subsequently referred to as ‘synapse’) firing once, on average. When few coincident synapses were stimulated, the EPSP in the stimulated distal branch was small and increased approximately linearly from the resting potential (−58 mV; similar to −57 mV measured in slices [36]) as the number of synapses increased. However, above 15 synapses, the duration of the EPSP increased dramatically and exhibited a flat top, hallmarks of a local regenerative NMDAR spike (**Fig. 1C**). We identified the presence of NMDAR spikes in a dendritic branch using a −30 mV voltage threshold criterion (**Fig. S1**). By stochastically varying the spatial and temporal patterns of synaptic input onto a dendritic branch, and measuring the occurrence of NMDAR spikes over 10 trials, it was possible to build an input-output (I-O) relationship for each individual branch. That is, the relationship between the number of stimulated synapses and the probability of generating an NMDAR spike (P(NMDAR spike)). **Fig. 1D** shows the fits of a sigmoid function (Materials and Methods) to the I-O relationships for all 28 terminal branches in the model (mean length = 72±52 µm). Between 10 and 25 temporally coincident synapses were required to trigger an NMDAR spike with a probability of 0.5 in the terminal branches. On average 18 synapses were required and this increased to 30 coincident synaptic inputs to reliably trigger an NMDAR spike on every trial. These values are in good agreement with experimental estimates from dendritic recordings [31] and glutamate uncaging experiments on L2/3 [35] and L5 neurons [36] in acute slices and previous modelling studies [29], [44]. Given that the average number of spines per terminal branch is approximately 140, assuming a spine density of 2 µm^−1^ [57], these simulations suggest that nearly coincident activation of a substantial fraction (13–20%) of the synapses on a terminal branch is required to activate a dendritic spike reliably, under quiescent conditions.

### Background excitatory synaptic input reduces the threshold for NMDAR spikes

We next examined how asynchronous background excitatory synaptic input affected integration in the tuft. We did not include background inhibition in these initial simulations, in order to study the effects of background excitatory inputs in isolation. We estimated the rate of background excitatory synaptic input *in vivo* from both anatomical and functional measurements. Tufted L5 pyramidal cells are innervated by ∼15,000 excitatory synaptic contacts (assuming 1 per spine), of which 3500 occur on the apical trunk and dendritic tuft in layers 1–3 [57], [58]. Since many of these contacts arise cortically from other L5 and L2/3 pyramidal cells, which typically fire at ∼1 Hz *in vivo* [49], [50], a L5 pyramidal cell is expected to experience a background quantal synaptic input rate of ∼1750 Hz on its apical dendritic tree, assuming a release probability of ∼0.5 per synaptic contact [59]. To mimic a modest level of background excitatory synaptic input, we randomly distributed 900–1500 quantal synaptic inputs over the apical tuft (including the Ca^2+^ hot zone; Blue region, **Fig. 1A**) and drove each synapse with a different 0.85 Hz Poisson train, resulting in a background synaptic input rate of 765–1250 Hz. These modest levels of uncorrelated background excitatory input rarely triggered spontaneous NMDAR spikes (<1% of trials, which were excluded from the analysis whenever possible), but did depolarize the dendritic membrane potential by 2–5 mV, within the range of experimental recordings [36], [47] (**Fig. 1E, F**) and generated voltage noise at the soma with a standard deviation of 0.86–1.26 mV.

We next examined how the branch I-O relationship was affected by background excitatory synaptic input. These simulations revealed that many fewer coincident synaptic inputs were required to trigger an NMDAR spike than for quiescent control conditions (**Fig. 1G**). On average, the number of synapses required to trigger an NMDAR spike with 50% probability fell from 18 to 6 in the presence of 1500 background synapses (**Fig. 1H**). A lower threshold for NMDAR spike generation and an increased in slope (gain) of the branch I-O relationship was also observed with background input when the time window for synaptic input was increased from 5 to 15 ms and the input frequency scaled down to maintain ∼1 quantal conductance per synapse (**Fig. 1I**). These simulations show that NMDAR spikes can be triggered reliably in terminal branches by much smaller numbers of nearly-coincident synaptic inputs (i.e. activation of 4–7% of the total synapses) in the presence of modest levels of background excitatory synaptic input, than under quiescent conditions.

### Background excitatory input decreases the number of stimulated branches required to trigger action potentials

**Figure 2:**
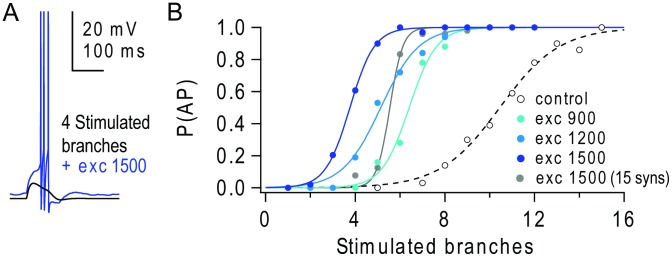
Background excitatory input reduces the number of nearly synchronously stimulated branches required to trigger action potentials. (**A**) Somatic voltage response to the activation of 4 apical branches stimulated with 30 nearly synchronous synapses each, in the absence (black) and presence (blue) of 1500 background excitatory synapses distributed on the apical tuft. (**B**) Probability of triggering action potentials (P(AP)) versus number of stimulated branches, in the absence (black open circles) and presence of different levels of background excitation (blue filled circles). Lines show fits to a sigmoid function. Grey line and circles show results for 15 synaptic inputs per branch and 1500 background excitatory synapses.

To investigate whether background excitatory synaptic input also affects the spike output of the pyramidal cell model, we determined the number of stimulated branches required to trigger somatic APs. To do this we randomly selected a group of N branches within our set of 28 terminal branches in the tuft and simultaneously stimulated each branch with 30 near-synchronous synaptic inputs (5 ms window) to ensure a high probability of triggering an NMDAR spike in each branch under all conditions (**Fig. 1H**). We then systematically altered the number of stimulated branches and determined whether the neuron fired APs or not. This rather artificial synaptic input configuration was chosen to quantify the impact of excitatory background input when multiple branches were stimulated. **Fig. 2A** shows an example where stimulation of 4 apical branches produced only a subthreshold somatic potential during quiescent control conditions. Systematically increasing the number of stimulated branches, while averaging across many combinations of branches to minimize branch specific effects, revealed that in the absence of background input 11 near-synchronous stimulated NMDAR spikes were required to trigger APs with 50% probability, and 15 were required to trigger an AP reliably (**Fig. 2B**).

By contrast, in the presence of background excitatory synaptic input, the relationship between the probability of triggering APs (P(AP)) and the number of stimulated dendritic branches exhibited a lower activation threshold and was much steeper (**Fig. 2A, B**). Indeed, the number of stimulated branches required to trigger an AP with 50% reliability was reduced from 11 to <4 for 1500 background excitatory synapses. The enhanced efficacy of NMDAR spikes was not dependent on the strong activation of individual branches, since reducing the number of synapses per stimulated branch from 30 to 15 still produced a leftward shift in the I-O relationship (**Fig. 2B**, grey). Thus, even the modest level of background excitatory input used here substantially increased the efficacy of NMDAR spikes in triggering APs. These results suggest that, in the absence of inhibition, modest levels of background excitatory synaptic input arising from ongoing network activity could increase the probability of NMDAR spikes occurring in the dendritc tuft and increase AP generation.

### Background excitatory input extends the temporal integration window in pyramidal cells

**Figure 3:**
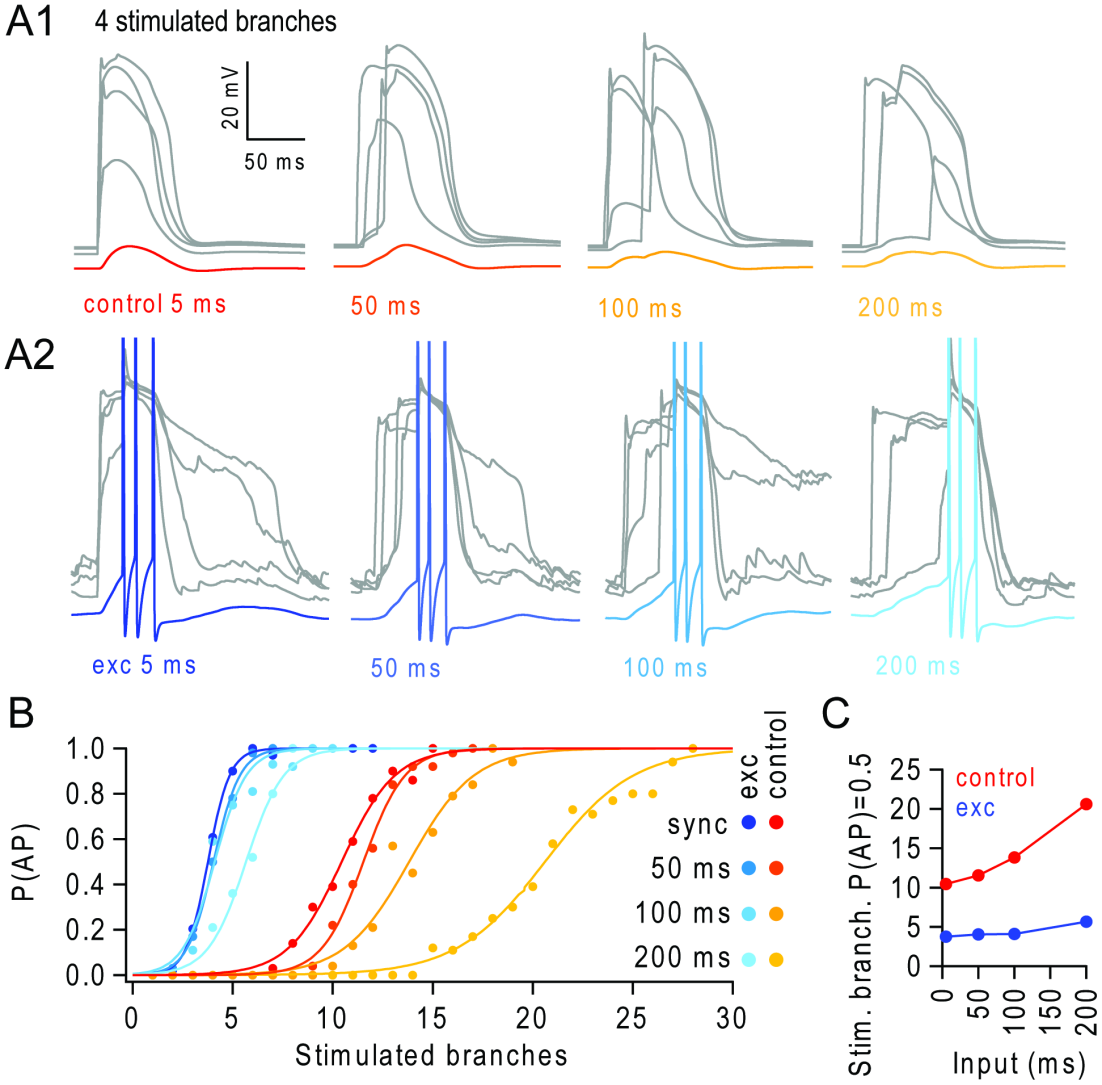
Background excitatory input extends the temporal integration of stimulated branches. (**A1**) Left panel: example voltage traces in 4 terminal apical branches during near synchronous branch activation (5 ms window, 30 synapses per branch; grey traces) and from the soma (red). Other panels show responses when stimulated branches are desynchronized in progressively larger temporal windows (50 ms, 100 ms, 200 ms; soma traces lighter shades of orange). (**A2**) Same as A1 but during background input from 1500 excitatory synapses, for near-synchronous branch stimulation (5 ms; dark blue) or desynchronized in progressively larger temporal windows (50 ms, 100 ms, 200 ms; soma traces lighter shades of blue). (**B**) Probability of action potentials (P(AP)) versus number of stimulated apical branches for different degrees of temporal dispersion during background activity from 1500 background excitatory synapses (blue lines) compared to control condition (red-yellow lines). (**C**) Number of stimulated branches required to trigger an AP with 50% probability (P(AP) = 0.5), for different levels of temporal dispersion in the stimulated branches (input window), in the absence (red) and presence of background excitatory input (blue).

To examine how the L5 pyramidal cell model integrated inputs on longer time scales than the nearly coincident synaptic input examined so far, we desynchronized the stimulation of different dendritic branches located over the apical tuft. To do this we stimulated each branch with a near-synchronous (5 ms) burst of synaptic input, but activated different branches at random times within a time window that ranged from 5 ms to 200 ms and examined how desynchronization of branch activation affected the probability of triggering APs. In the absence of background excitatory input, 4 stimulated branches produced only a subthreshold response (**Fig. 3A1**). The average number of stimulated branches required to trigger an AP with 50% reliability gradually increased from 11 to 21 as stimulation of the branches was desynchronized from 5 ms to 200 ms (**Fig. 3B**). This result suggests that under quiescent conditions, L1 synaptic input would need to be both synchronous and strong enough to trigger NMDAR spikes in a substantial fraction of the terminal branches in order to produce an AP.

By contrast, background excitatory synaptic input substantially reduced the number of stimulated branches required to trigger APs (e.g. 4 branches, **Fig. 3A2**) and asynchronous branch activation within a 100 ms window became just as effective at triggering APs as coincident activation of the dendritic branches (**Fig. 3B**). The number of stimulated branches required to trigger an AP only slightly increased when branches were stimulated over a 200 ms window (**Fig. 3B, C**). The longer the time window over which the NMDAR spikes occurred, the larger the absolute reduction in the number of stimulated branches required to trigger APs with background input, compared to control conditions (**Fig. 3C**). These results suggest that, in the presence of modest levels of background excitatory input, clusters of synchronous synaptic inputs could be integrated over timescales that are considerably longer than both the membrane time constant of L5 pyramidal cells (∼10–20 ms [60]) and the NMDAR-mediated synaptic integration window in individual terminal branches of L2/3 pyramidal cells recorded in acute slices using glutamate uncaging (∼10 ms integration window [**35**]), where background network activity and inhibition were largely absent.

### Background excitatory synaptic input enables integration of spatially and temporally distributed excitatory synaptic input

**Figure 4:**
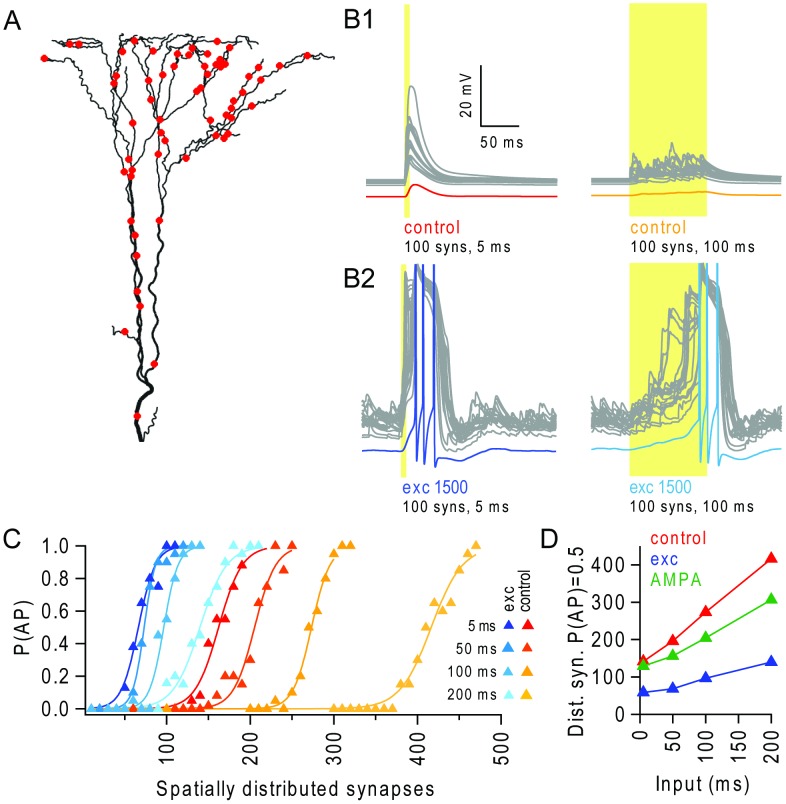
Background excitatory input enables integration of spatially distributed synaptic input over extended temporal windows. (**A**) Apical tuft with an example distribution of 60 synaptic inputs (red dots). (**B1**) Left: example voltage traces from all terminal apical branches (grey traces, N = 28) and the soma (red) in response to stimulation of 100 spatially distributed synaptic inputs at 200 Hz for 5 ms, during control conditions. Right: example trial for 100 ms stimulation window. (**B2**) Same as B1 in the presence of background activity from 1500 excitatory synapses. Somatic voltage traces are in blue and are truncated at +10 mV. (**C**) Probability of triggering action potentials (P(AP)) versus number of stimulated synapses, spatially distributed over the apical tuft. Synapses were driven with random trains with a window of 5 ms, 50 ms, 100 ms and 200 ms, and the frequency was scaled to maintain ∼1 quantal conductance per synapse, in the absence (red-yellow traces) and presence of background excitatory input (blue traces). (**D**) Number of distributed synapses required to trigger an AP with 50% probability (P(AP) = 0.5), for different levels of temporal dispersion in the stimulus evoked synapses (input window), in the absence (red) and presence of background excitatory input (blue), and during AMPAR-only background synaptic inputs which depolarized the dendrites to comparable levels (green).

To investigate how background excitatory synaptic activity affects the integration of spatially distributed input, we examined the I-O relationship of the model when stimulus-evoked synaptic input was randomly distributed across the entire apical tuft (**Fig. 4A**), rather than being clustered on selected branches. In the absence of background network activity, near synchronous activation (5 ms duration random burst at 200 Hz) of 100 excitatory quantal synapses typically generated subthreshold responses (**Fig. 4B1**), while 160 synapses triggered an AP with a 50% probability (**Fig. 4C**). By contrast, 100 near-synchronous synapses triggered numerous NMDAR spikes and somatic APs in the presence of 1500 background excitatory synapses (**Fig. 4B2**). Indeed, only 60–70 nearly synchronously activated synapses were required to reach P(AP) = 0.5 in the presence of background excitatory input (**Fig. 4C**). When the temporal coincidence of the stimulus-evoked input was relaxed from 5 ms to 50 ms (and the input frequency scaled down to ensure the same total amount of excitation per synapse), the number of spatially distributed inputs required to reach P(AP) = 0.5 rose to >200 in the absence of background input (**Fig. 4C**). However, 60–70 spatially and temporally distributed synapses were still sufficient to reach P(AP) = 0.5 in the presence of background excitatory input, only marginally more than for coincident input (**Fig. 4C**). Temporally dispersing the synaptic input further to 100 ms (**Fig. 4B1,B2**) or 200 ms required a greater number of synaptic inputs, but the number of synapses required in the presence of background excitatory input remained approximately 3-fold less than in its absence (**Fig. 4C,D**). These simulations show that modest levels of background excitatory synaptic input can extend both spatial and temporal integration in a L5 pyramidal cell model, when compared to quiescent conditions.

### Synaptic integration during balanced background excitatory and inhibitory synaptic input

So far we have examined how synaptic integration in the apical tuft of a L5 pyramidal cell model is altered by background excitatory synaptic input. However, under physiological conditions ongoing cortical network activity consists of both excitatory and inhibitory conductances in an approximately balanced configuration [15], [16], [18]. Although our understanding of cortical inhibition is far from complete, our knowledge of the properties of inhibitory synaptic inputs onto pyramidal cells has expanded recently, as a result of a number of *in vitro* and *in vivo* studies [48], [61]–[66]. These studies show that Somatostatin (SOM) expressing interneurons (which include Martinotti cells) innervate the dendritic tuft of L5 pyramidal cells with GABA_A_ receptor mediated synaptic input [63], while neurogliaform (NGF) cells, which have dense axonal plexi, form numerous mixed GABA_A_ and GABA_B_ receptor mediated synapses [62]. These dendrite targeting inhibitory interneurons [62], [63], [67] have been shown to powerfully control dendritic gain [68] and neuronal firing during behaviour [48]. Moreover, distributed inhibition has been shown to be particularly effective in shunting excitation in branched structures such as the dendritic tuft [69].

To examine how synaptic integration in L5 pyramidal cells might operate under more physiological conditions, when network activity delivers balanced excitatory and inhibitory background synaptic input, we added 60 SOM-like GABA_A_ receptor mediated synapses and 13 neurogliaform (NGF) cell-like mixed GABA_A_/GABA_B_ synapses onto the apical dendritc tree. These inputs were driven at firing rates reported for passive touch whisker experiments in awake animals and are likely to reflect the upper end of the rates observed during active touch, which fall to about half this level on average [48]. The GABA_A_ receptor component had a fast rise and a decay time of 10 ms [66], while the GABA_B_ receptor-mediated K^+^ conductance had a slow rise and decay time of 50 and 80 ms, respectively, with peak values of 0.5 nS and 0.06 nS and an activation delay of 10 ms [70] (**Text S1 Supporting information**). This produced somatic IPSPs with properties comparable to those measured experimentally [61], [62] (**Fig. S2**). Although the inhibitory synapses were fewer in number than the excitatory synapses, their higher firing rate and slower kinetics effectively counterbalanced the AMPAR component of the 1500 excitatory synapses, producing a time averaged GABAR/AMPAR conductance ratio of 1.5, comparable to that estimated from synaptic currents [16], [71]. We refer to this experimentally constrained configuration of asynchronous background excitation and inhibition as “balanced background synaptic input”.

**Figure 5:**
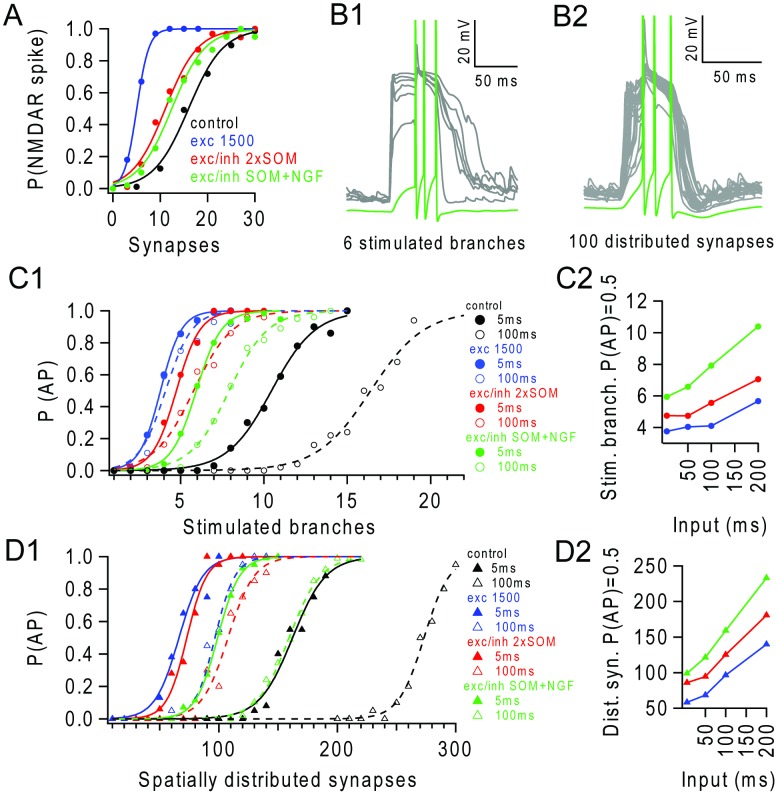
Background inhibition modulates spatio-temporal integration. (**A**) Probability of an NMDAR spike (P(NMDAR spike)) occurring in a terminal branch versus number of nearly synchronous stimulus evoked synapses during control (black), in presence of background activity from 1500 excitatory synapses (blue) and during mixed excitatory and inhibitory background (gGABA/gAMPA ratio of 1.5) provided by 130 pure GABA_A_ receptor (SOM-like) synapses firing random trains at 3 Hz (2×SOM, red) or by a combination of 65 SOM-like synapses and 13 NGF-like synapses providing both GABA_A_ and GABA_B_ receptor-mediated inhibition, firing random trains at 14 Hz (SOM+NGF, green). (**B1**) Example of voltages in 6 apical branches (grey) and soma (green) during nearly synchronous stimulation of 6 branches in the presence of ballanced background input (SOM+NGF). (**B2**) Example of voltages in all apical branches (grey) and soma (green) during nearly synchronous stimulation of 100 spatially distributed stimulus evokes synaptic inputs in the presence of balanced background input (SOM+NGF). (**C1**) Probability of triggering APs (P(AP)) versus number of nearly synchronously stimulated branches (5 ms, filled circles, solid lines) or asynchronously stimulated branches during a 100 ms window (open circles, dashed lines) for conditions in (A). (**C2**) Increase, from the nearly synchronous condition, in the number of stimulated branches required to trigger an AP (P(AP) = 0.5) with different levels of temporal dispersion (input) for conditions in (A) excluding control. (**D1**) P(AP) versus number of stimulated synapses for spatially distributed input over the apical tuft for conditions in (A). Synapses activated with random trains at 200 Hz for 5 ms (solid markers) or 10 Hz for 100 ms (empty markers). (**D2**) Number of spatially distributed synapses required to trigger an AP (P(AP) = 0.5) for different levels of temporal dispersion (input window), for conditions in (A).

When the balanced background synaptic input included SOM-type and NGF (SOM+NGF) mediated inhibition, the NMDAR spike threshold was reduced compared to quiescent conditions, but not as strongly as during background excitation alone (**Fig. 5A**). The fact that the NMDAR spike threshold was similar for SOM+NGF and for a SOM-only case where the number of SOM-inputs was doubled (2×SOM), to maintain the same total GABA_A_ receptor mediated conductance, suggests that the slow low-amplitude GABA_B_ component and the GABA_A_ component are equally effective at inhibiting NMDAR spikes (**Fig. 5A**). Near-synchronous activation of 6 branches triggered an AP burst (P(AP) = 0.5) during SOM+NGF mediated inhibition (**Fig. 5B1**). However, SOM+NGF mediated inhibition was more effective at counteracting the background excitation-mediated increase in AP probability in response to multiple asynchronously activated branches than the 2×SOM input alone (**Fig. 5C1, C2**).

For spatially distributed synaptic input, the number of nearly synchronous inputs required to trigger APs was only marginally higher for 2×SOM-only balanced background inhibition than background excitation alone (**Fig. 5D1, D2**). However, for combined SOM+NGF inhibition, the number of synapses required to trigger an AP burst increased, indicating that the presence of the slow inhibitory component was effective at lowering dendritic gain for spatially distributed synaptic inputs (**Fig. 5D1, D2**). The fact that NMDAR spikes were evident in the voltage recordings from the terminal branches indicates that nonlinear dendritic integration underlies the AP output during distributed input in the presence of SOM+NGF inhibition (**Fig. 5B2**). Balanced background inhibition mediated by SOM+NGF was also effective at counteracting asynchronous inputs, increasing the number of spatially distributed inputs requited to trigger an AP burst with P(AP) = 0.5, from 100 with background excitation alone to 170 for a temporal window of 100 ms (**Fig. 5D1,D2**). Nevertheless, this was still far lower than under quiescent condition when 273 synapses were required. These simulations show that GABA_A_ and GABA_B_ receptor-mediated inhibitory conductances can counteract the effects of background excitatory input on synaptic integration in the dendritic tuft by raising NMDAR spike threshold and lowering the dendritic gain. Our simulations predict that during periods of lighter dendritic inhibition, for example when both sensory and motor systems are engaged [10], [48], the presence of balanced background synaptic input increases dendritic gain and extends the spatio-temporal integration properties of L5 pyramidal cells.

In the subsequent sections we investigate the mechanisms underlying network activity-dependent spatio-temporal integration in L5 pyramidal cells.

### Mechanisms underlying the changes in spatio-temporal integration during background excitatory input

Several dendritic mechanisms could be involved in the network activity-dependent changes in synaptic integration we observe, but two are likely to be particularly important. Background excitatory inputs activate glutamatergic synapses that: 1) depolarize the dendrite (**Fig. 1F**) and 2) generate glutamate-bound but mostly silent NMDARs over the dendritic tree. Depolarization [33], glutamate spillover [72] and glutamate-bound NMDARs from paired-pulse synaptic activation [73], [74] have been shown to facilitate the generation of local NMDAR spikes in basal dendrites. In contrast to these relatively local effects, background network activity confers a spatially distributed NMDAR conductance that could provide an additional nonlinear resource for regenerative activity across the dendritic tree.

Since the threshold of NMDAR spikes is voltage-dependent [33], we first examined the effect of the depolarization induced by excitatory background activity on NMDAR spike properties. To do this we applied spatially distributed current injections (**Fig. S3A1**) that depolarized the apical tree to a level comparable to that during background excitatory synaptic input (**Fig. S3A3, inset**). Current injections accounted for a substantial part of the change in the single-branch I-O relationship observed with background input (**Fig. S3A2**). However, when spatially distributed AMPAR-only synapses were used to depolarize the apical tree to a comparable level of depolarization obtained with 1500 AMPAR/NMDAR synapses, they were less effective at altering the branch I-O relationship, due to the shunt introduced by these conductances (**Fig. S3A2**). Similarly, AMPAR-only background activity that generated comparable dendritic depolarization only accounted for a fraction of the changes observed in the neuronal I-O relationship in response to clustered (**Fig. S3A3**) and spatially distributed inputs (**Fig. 4C**). Moreover, current injections that produced depolarisations comparable to that obtained during background synaptic input at the Ca^2+^-spike initiation zone and at the soma recovered only a small part of the leftward shift in the neuronal I-O relationship (**Fig. S3B, C**). These results show that, while depolarization is important in determining NMDAR spike threshold [33], when depolarization is mediated by AMPAR-only background synaptic conductance it does not fully account for the lowering of NMDAR spike threshold or the increased efficacy of apical input in triggering APs during background input. This suggests that the spatially distributed NMDAR conductance arising from background network activity is also playing a key role in synaptic integration.

### NMDAR component of background synaptic input extends the duration of NMDAR spikes

**Figure 6:**
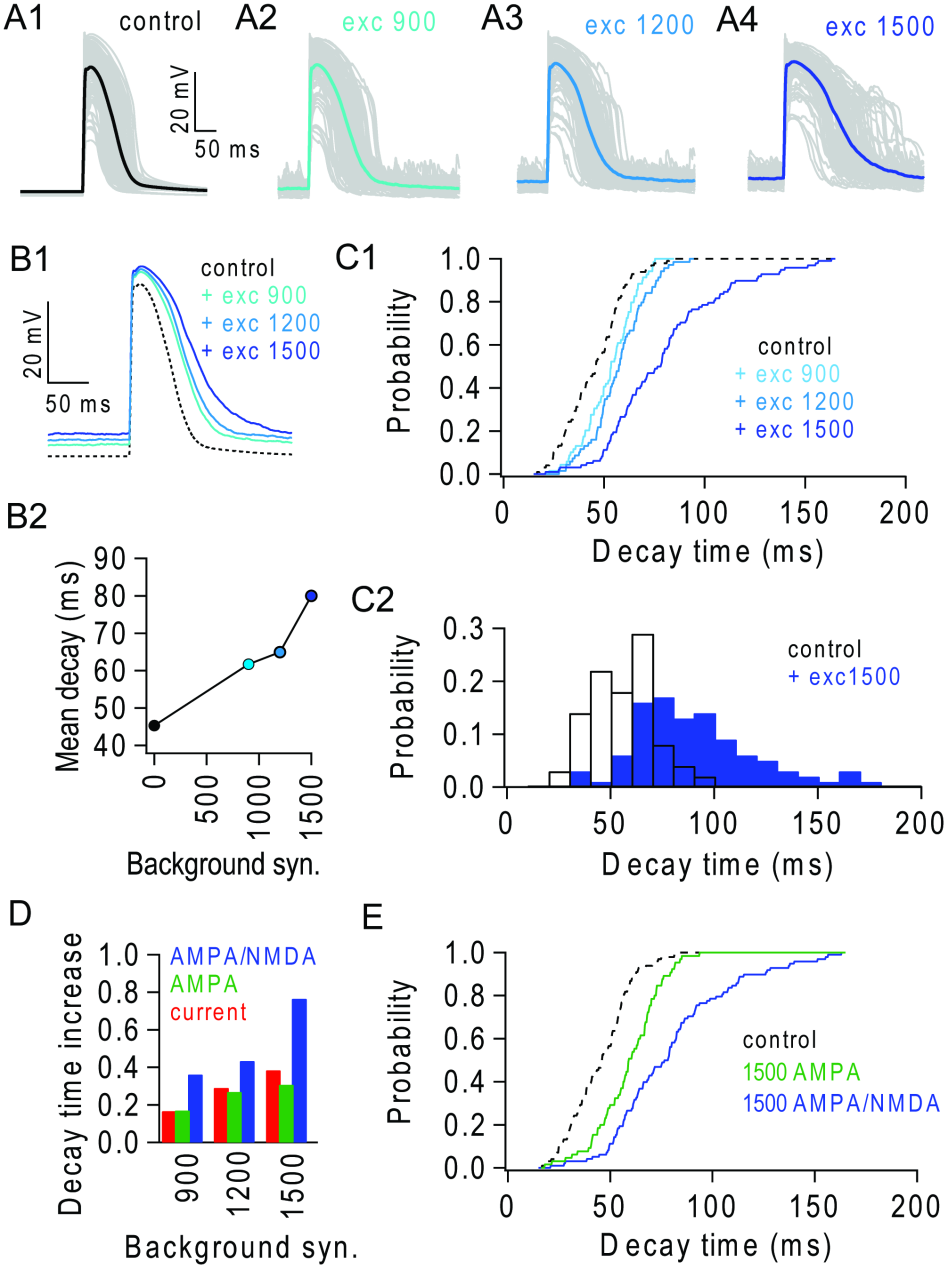
Background excitatory input extends the duration of NMDAR spikes. (**A1–4**) Dendritic NMDAR spikes triggered in different terminal branches (30 synapses; 100 trials) in the absence (control) and presence of 900, 1200 and 1500 background excitatory synapses. Single trials (grey) and average (solid colour). (**B1**) Average NMDAR spikes in (A) overlaid. (**B2**) Average decay time (37% of peak) of NMDAR spikes in (A). (**C1**) Cumulative distributions of decay times for control and different levels of background input. (**C2**) NMDAR spike decay time distribution in the absence (black) and presence (blue) of 1500 background synapses. (**D**) Fractional increase in average NMDAR spike decay time for excitatory background synapses containing AMPAR/NMDARs (blue) and equivalent dendritic depolarization obtained with background AMPAR-only synapses (green) or current injection (red). (**E**) Cumulative distributions of NMDAR spike decay times during depolarization mediated by AMPAR-only (green) and mixed AMPAR/NMDAR (blue) background synaptic input.

Comparison of the properties of NMDAR spikes in apical branches during different levels of background excitatory synaptic input revealed that the duration of NMDAR spikes systematically increased with the amount of background activity (**Fig. 6A1–4**). The mean duration increased from 45 ms in the absence of background input to 79 ms in the presence of 1500 background synapses (**Fig. 6B1–2**). Examination of the distribution of spike duration revealed an increased dispersion and some very long events (>150 ms; **Fig. 6C1–2**). Depolarization with current injections into the apical tuft accounted for part, but not all, of the increase in NMDAR spike duration and the contribution of depolarization became smaller at higher background rates (**Fig. 6D**). Replacing the current injections with AMPAR synaptic conductances accounted for less than half of the shift in the decay time distribution with 1500 background excitatory synapses (**Fig. 6D–E**). These results are consistent with the idea that both depolarization and the presence of glutamate-bound NMDARs over the dendritic tree contribute to the lengthening of NMDAR spikes during background input.

### Propagation and multiplication of dendritic regenerative events during background excitatory synaptic input

**Figure 7:**
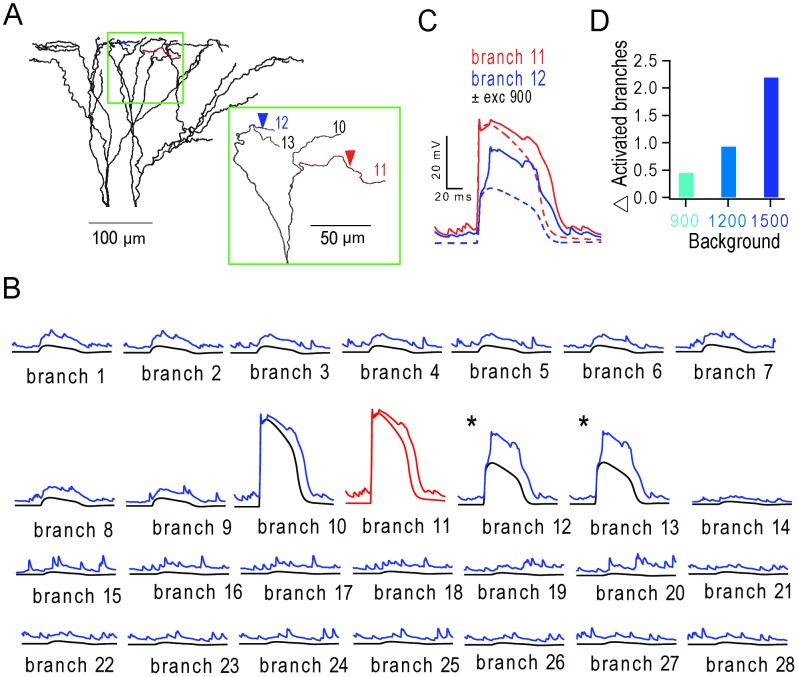
Stimulation of a dendritic branch triggers regenerative potentials in neighbouring branches during background synaptic input. (**A**) Apical tuft with inset showing branch 11 (red) stimulated with 30 synaptic inputs (5 ms window) and branch 12 (blue) that receives no stimulus evoked input. Inset: enlarged tuft region with overlapping branches removed for clarity. (**B**) Membrane voltage of all 28 terminal apical branches during activation of branch 11 (red trace) in the presence (upper trace) and absence (lower trace) of distributed background synaptic activity from 900 excitatory inputs. Asterisks denote additional regenerative events triggered in branches 12 and 13 in the presence of background activity. (**C**) Voltage in branch 11 (red) and branch 12 (blue) with (solid lines) and without (dashed lines) background activity. (**D**) Average number of additional regenerative events triggered in neighbouring branches (identified using a 13 mV increase above level observed in the absence of background input) during different levels of background excitatory input (10 trials per branch, per condition).

The finding that spatially distributed NMDAR conductance arising from background excitatory synaptic input contributes to the lengthening of dendritic spikes, suggests that glutamate-bound NMDARs in the vicinity of stimulated branches are recruited by depolarization. To investigate the extent of this effect, we monitored the voltage in each terminal branch, while triggering an NMDAR spike in only one branch at a time, with and without the background input (**Fig. 7A** **; Movie S1**). In the absence of background input, triggering an NMDAR spike in branch 11 with 30 nearly synchronous synaptic inputs produced a large voltage depolarization (**Fig. 7B**, lower red trace) that spread into the neighbouring branch (10) producing a similar voltage profile (**Fig. 7B**, black trace). However, the voltage in more distant branches (12 and 13) was markedly attenuated. When the same branch was triggered in the presence of background excitatory synaptic input (upper red trace) both the duration of the NMDAR spikes in branches 10 and 11 were extended and an additional regenerative event was observed in branch 12 and branch 13 (blue traces), suggesting that glutamate-bound NMDARs had become unblocked in neighbouring branches. Since it is difficult to distinguish between regenerative events and passive voltage propagation in the presence of background input (**Fig. 7C**), we used a more stringent voltage threshold criterion to identify regenerative events in neighbouring branches (peak_background_−peak_control_≥13 mV). The average number of additional activated branches ranged from 0.46 with 900 background excitatory inputs, to 2.19 with 1500 background excitatory inputs (**Fig. 7D**). These results show that additional regenerative events can be generated in neighbouring branches during background synaptic activity (**Movie S1**). This nonlinear dendritic amplification potentially explains why fewer stimulated branches are required to trigger APs in the presence of background excitatory synaptic input (**Fig. 4A**).

### Lowered threshold, extended duration and spread of regenerative NMDAR events during background excitatory synaptic input in a passive L5 pyramidal cell model

**Figure 8:**
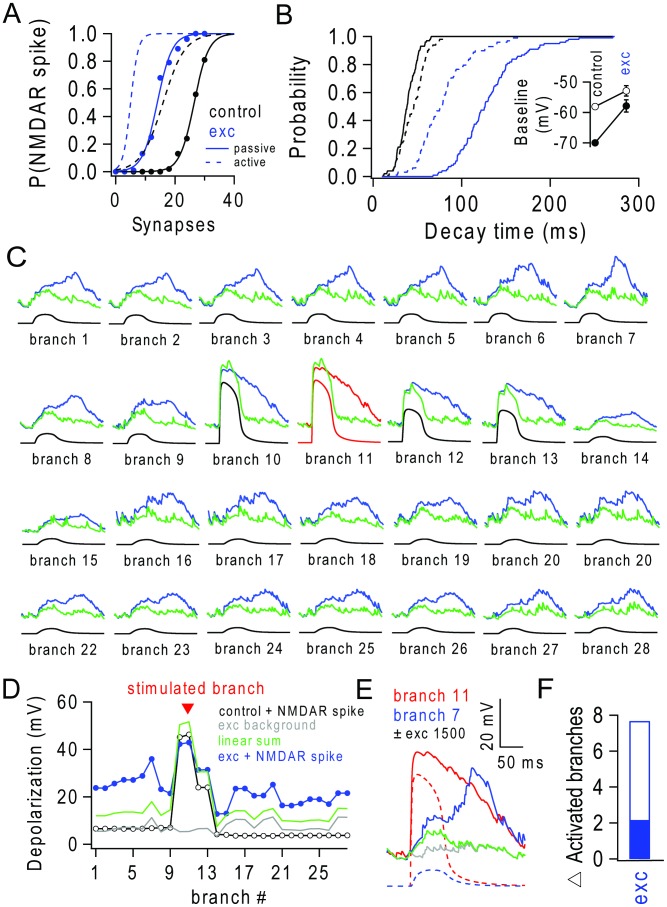
Background excitatory input lowers threshold, extends duration and regeneration of NMDAR spikes in a passive model. (**A**) Probability of triggering an NMDAR spike (P(NMDAR spike)) in terminal branches versus number of nearly synchronous stimulated synapses in a passive model (solid lines), where all non-synaptic active dendritic conductances have been removed, in the absence (black) and presence of 1500 background excitatory synapses (blue). Original model shown for comparison (dashed lines). (**B**) NMDAR spike decay time distribution (N = 100, triggered with 30 synapses each) lines as for (A). Excitatory decay times distribution during control and during background activity. Inset, mean voltage (±SD) in randomly selected terminal branches (N = 100) with and without background activity in passive (filled symbols) and original model (open symbols). (**C**) Membrane voltage traces from all 28 terminal apical branches (as in **Fig. 7**) in the passive model for a single trial, during nearly synchoronous activation of branch 11 (red traces) in the presence (upper trace; blue) and absence (lower trace; black) of background activity from 1500 excitatory inputs. Linear sum of the depolarization during control and during background input only, shown in green. (**D**) Peak depolarization induced by an NMDAR spike in branch 11 versus terminal branch number in absence (black) and presence (blue) of background activity. Peak depolarization during background activity alone shown in grey and peak value of linear sum of control during stimulation of branch 11 and background-only shown in green. (**E**) Voltage traces from branch 11 (red) and branch 7 (blue) in the absence (dashed lines) and presence of 1500 background excitatory inputs (solid lines). Grey and green trace as in (D). (**F**) Number of additional branches activated during background input when a single branch is stimulated (empty bar). This was estimated by stimulating the terminal branches in turn for 10 trials each and applying the 13 mV criterion, which identified regenerative peaks, although the number of branches exhibiting regenerative events is probably overestimated due to passive spread of voltage. Solid bar shows original model for comparison.

L5 pyramidal cells contain several nonlinear voltage-dependent dendritic conductances that could interact with synaptic input in complex ways. We therefore examined whether any of the basic changes in synaptic integration we observed in the presence of background excitatory input occurred in a passive L5 model, where all the non-synaptic nonlinear conductances were replaced by a uniform passive leak conductance. Although there were some differences between the resting potential of this passive model compared to the active model (the passive model is more hyperpolarized) asynchronous background synaptic input lowered NMDAR spike threshold and elongated the duration of NMDAR spikes (**Fig. 8A, B**). These results confirm that synaptic AMPAR and NMDAR conductances are sufficient to change NMDAR spike threshold and duration during asynchronous excitatory background synaptic input.

We also examined whether propagation of regenerative events into neighbouring dendritic branches that we observed during background excitatory input could be generated by synaptic AMPARs and NMDARs in the passive model. **Fig. 8C** shows an example where we stimulated a branch and examined the response in the other distal branches of the tuft. Regenerative events in neighbouring branches were less evident, nevertheless the voltage depolarization in neighbouring dendritic branches during background input was larger than the linear sum of the depolarizations observed with the stimulus alone and the background input alone (**Fig. 8C, D**). This suggests that, in the presence of background excitatory input, the spread of voltage depolarization from the stimulated branch has a nonlinear regenerative component. Indeed, the duration of the voltage depolarisations in neighbouring branches also increased and some continued to build up well after the peak depolarization in the stimulated branch (**Fig. 8E**). As for the full active model, regenerative events were observed in neighbouring branches when a single branch was stimulated during background input (**Fig. 8F**).

These results suggest that synaptic AMPARs and NMDARs activated by background excitatory input can mediate active propagation into neighbouring branches, under certain conditions. However, the presence of dendritic Na^+^ and Ca^2+^ conductances in real cells [75] and in the original model are likely to facilitate this process. The spread of NMDAR-mediated regenerative events across branches and the increased decay time are likely to be interlinked: spread of voltage into neighbouring branches recruits NMDAR conductance activated by background input, potentially triggering a regenerative event. Recruitment of NMDAR conductances will help sustain the depolarization, lengthening the NMDAR spike duration, which in turn propagates more effectively through the dendritic tree. Simulations show that during background activity, a few synchronous distributed inputs can trigger an ‘avalanche’ of activity in multiple branches that feeds and sustains the depolarization for more than one hundred milliseconds. These events, through the recruitment of Ca^2+^ currents in the trunk, can then initiate somatic APs (See **Movie S2**). Propagation of voltage and activation of spatially distributed NMDAR conductance in neighbouring branches can therefore account for the increased efficacy of NMDAR spikes and the extended spatio-temporal integration we observe in the L5 pyramidal cell model, during background excitatory synaptic input.

### Effect of inhibition on NMDAR spike initiation, duration and regeneration in the tuft

**Figure 9:**
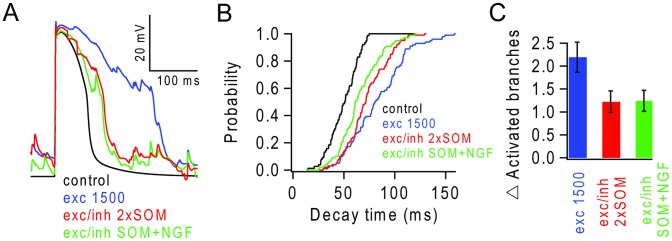
Background inhibition modulates NMDAR spike duration and regeneration during background excitatory input. (**A**) Single trial of voltage response of apical branch triggered by nearly synchronous activation of 30 synapses (5 ms) during control (black), in presence of background activity from 1500 excitatory synapses (blue) and during mixed excitatory and inhibitory background (gGABA/gAMPA ratio of 1.5) provided by 130 pure GABA_A_ receptor mediated synapses (2×SOM) firing at 3 Hz (red) or by a mixture of 65 SOM-like synapses and 13 NGF-like synapses providing both GABA_A_ and GABA_B_ receptor mediated inhibition firing at 14 Hz (SOM+NGF, green). (**B**) Cumulative distributions of NMDAR spike decay times for conditions in (A). (**C**) Additional branches activated during stimulation of a single branch for conditions in (A) (10 trials per branch, per condition).

Having established that depolarization and spatially distributed NMDAR conductances are principally responsible for extending spatio-temporal integration during background excitatory synaptic input, we next examined how inhibition counteracts these effects. Comparison of individual NMDAR spikes during background excitatory synaptic input and during balanced background synaptic input, showed that the presence of SOM+NGF inhibition had little effect on their amplitude but did shorten their duration [44] (**Fig. 9A**). Inspection of the NMDAR spike decay time distribution showed that inhibition truncated the longer NMDAR spikes, making the distribution less skewed (**Fig. 9B**). Moreover, SOM+NGF inhibition was more effective than 2×SOM at truncating NMDAR spikes, explaining why GABA_B_ receptor inhibition was effective in modulating temporal integration (**Fig. 5**). However, NMDAR spike duration was still substantially longer in the presence of SOM+NGF based balanced background synaptic input than under quiescent conditions (Kolmogorov–Smirnov test, P<0.05; **Fig. 9B**).

We next examined the effect of background excitation and inhibition on the spread of NMDAR spikes into neighbouring branches. The average fractional increase in the number of branches exhibiting regenerative potentials was reduced from 2.19 to 1.2 when inhibition was added to background excitation (**Fig. 9C**). Interestingly, SOM+NGF and 2×SOM inhibition were equally effective, consistent with their comparable effects on NMDAR spike initiation (**Fig. 5A**). These simulations show that dendritic inhibition counteracts the effects of background excitatory input by increasing the threshold for NMDAR spikes and reducing spike duration and spread in neighbouring branches (see **Movie S1 and S2**).

## Discussion

Our simulations suggest that the background synaptic input arising from the ongoing cortical network activity observed *in vivo*, can extend the spatial and temporal properties of dendritic integration in L5 pyramidal cells. Spatio-temporal integration depends on background network activity, because it introduces spatially distributed synaptic NMDAR conductance over the dendritic tree and depolarizes the dendrites. Increasing the background excitatory synaptic input to levels expected *in vivo* has three main effects: 1) the number of coincident synaptic inputs required to trigger an NMDAR spike in a branch is reduced; 2) the duration of NMDAR spikes is increased, and 3) NMDAR-mediated regenerative events spread and trigger additional regenerative events in neighbouring dendritic branches. These mechanisms markedly reduce the number of spatially distributed synapses or stimulated terminal dendritic branches required to trigger APs. Inclusion of fast (GABA_A_) and slow (GABA_B_) receptor-mediated inhibitory conductances in the background synaptic input in an approximately balanced excitatory/inhibitory configuration only partially counteracted these effects. Our results show that *in vivo*-like balanced background input could enable L5 pyramidal cells to integrate spatially distributed and/or temporally dispersed synaptic inputs onto the tuft more effectively. Our findings suggest that the activity state of the cortical network can dynamically control the integrative properties of L5 pyramidal cells by adjusting the density of glutamate-bound NMDARs on the dendritic tree. This prediction has important implications for cortical processing.

### Mechanisms underlying dendritic integration in active networks

Our simulations show that spatially distributed synaptic NMDAR conductances arising from ongoing network activity extend spatial and temporal integration in the dendritic tuft of L5 pyramidal cells. This network activity-dependent nonlinear dendritic mechanism is distinct from the well-documented effects of noise and linear synaptic conductances on neuronal I-O relationships [20], [23]–[25], [27]. While AMPAR-mediated conductances do contribute to integration, they summate poorly during asynchronous input due to their rapid decays (τ = 2 ms), producing a mild dendritic depolarization, voltage noise and reduced membrane resistance [19]. By contrast, the slow kinetics of NMDARs (τ = 70 ms) [54], [76] summate effectively, allowing background network activity to set the density of NMDAR conductance on the dendritic tree. The dynamic nature of the spatially distributed NMDAR conductance contrasts with the properties of other spatially distributed voltage-dependent dendritic conductances (e.g. Na^+^, Ca^2+^ and K^+^ channels), which are typically modulated slowly, through plasticity [77]–[79] and homeostatic mechanisms, although the effective number of channels available could change rapidly with voltage due to inactivation.

Several properties of the GluN2A/B containing NMDARs present in L5 pyramidal cells are important for dendritic integration in the presence of background synaptic activity. Their strong block by Mg^2+^ below −60 mV [80], [81] prevents their involvement in signalling except when the dendritic branch on which they are located is depolarized. This, together with their high affinity for glutamate, means that GluN2A/B-containing NMDARs can remain glutamate bound and ‘silent’, yet primed and ready to contribute current if the voltage of the dendritic branch depolarizes [73], [74]. The steep voltage dependence of the NMDAR conductance introduces a highly nonlinear threshold, enabling regenerative depolarization, while the slow kinetics of the conductance extends the time course of local EPSPs beyond the local effective membrane time constant of the fine dendritic branches [31], [56]. Our simulations show that these properties of NMDARs enable them to extend spatial and temporal integration in L5 pyramidal cells during *in vivo*-like network activity. Moreover, our control simulations indicate this effect is robust across a range of NMDAR kinetics: the slower the decay of the synaptic NMDAR conductance the longer the absolute window for temporal integration in the absence of other counteracting conductances (**Fig. S4**). However, faster NMDAR kinetics would reduce the impact of background synaptic input unless there was a proportionally higher background input rate or a larger NMDAR/AMPAR peak amplitude ratio to maintain the same time-averaged NMDAR conductance. Another key property of NMDARs that sets them apart from nonlinear dendritic Na^+^ conductances [82] is the fact that they remain active during bursts of backpropagating APs and the sustained depolarisations during up-states [83].

By increasing the gain of dendritic integration, background excitation could trigger an unconstrained positive feedback loop, integrating over longer timescales and resulting in prolonged global activation of the dendritic tuft. In practice, cortical microcircuits operate in a balanced configuration with inhibition closely tracking excitation [16], [18], [48], [71], [84], [85]. Indeed, strong inhibition targeted to the tuft [43], [48] may be required to dampen excessive NMDAR excitation during highly active network states and prevent dendritic ‘chain reactions’ from reaching critical levels. Consistent with this, recent experimental evidence shows that apical inhibition has a stronger effect on the I-O relationship of CA1 pyramidal neurons than somatic inhibition [68]. The apical dendrites of pyramidal cells receive inhibition from SOM expressing interneurons, which include Martinotti cells [48]. SOM-expressing interneurons respond to synaptic inputs from neighbouring pyramidal cells and deliver fast GABA_A_ receptor mediated inhibition to the tuft region [63]. Interestingly, *in vivo* recordings show that SOM cell firing is suppressed for about 1 s during active whisker touch behaviour, suggesting that the tuft region is disinhibited during this behaviour [48]. Consistent with this, the G_exc_/G_inh_ ratio is higher during *in vivo* up-states [13], and engaging the motor and sensory circuits together during whisker touch behaviour results in global tuft activation [10]. Our simulations, which reproduced the excitation/inhibition balance and the firing rates of SOM and NGF cells measured in awake animals [48], show that inhibition reduces NMDAR spike threshold. However, our simulations of asynchronous background inhibition cannot rule out the possibility that fast feed-forward inhibition could veto NMDAR spike generation more effectively if it is present in L1 and L2 and targeted to the same branches as the excitation. Our simulations suggest that slow GABA_B_-mediated inhibition arising from NGF cells [61], [62], [65] is particularly effective at truncating NMDAR spikes and reducing dendritic gain. This, together with the fact that the firing rate of NGF cells is elevated during active touch [48], suggests that NGF mediated inhibition may be particularly well placed to control spatio-temporal integration in the tuft (**Fig. 5**). Such co-variation of background excitatory and inhibitory conductances [85] in recurrent cortical networks [43] is likely to extend the dynamic range over which pyramidal cells can operate by adjusting their integrative properties to match the excitatory drive. These findings extend previous work showing that inhibitory conductances can terminate NMDAR spikes [44] by showing how background inhibition arising from specific interneuron types could counteract the effects of background excitation and shape spatio-temporal integration in L5 pyramidal cells. Our results suggest that during active whisking the lowered level of inhibition [48], could promote NMDAR-mediated distributed signalling, while strong dendritic inhibition could keep it in check at other times, thereby preventing runaway excitation.

### Potential interactions between background synaptic input, NMDAR spikes and voltage-gated dendritic conductances

The dendrites of pyramidal cells contain many different voltage-gated conductances [86]. While it was not possible to explore all possible interactions between background input and the 9 conductances in our model, the simulations we carried out do point to some general principles of interaction and highlight specific conductances that could play a key role in shaping the properties of spatiotemporal integration. At the most basic level, some excitatory dendritic conductances in the model such as the Na^+^ and Ca^2+^ conductances clearly aid background induced regenerative activity (**Fig. S5**). Indeed, we found that Ca^2+^ spikes occur in the tuft during strong tuft stimuli, and help to electrically couple the tuft to the soma, as found experimentally [37]. By contrast, increasing I_h_ reduced the NMDAR spike threshold in the presence of background input, but had negligible effect on NMDAR spike duration (**Fig. S5**). Indeed, more complex effects are seen when conductances have activation and/or inactivation properties with significant voltage dependences around −55 mV, because background synaptic input depolarizes the dendrite by several millivolts compared to the quiescent condition. For example, transient A-type K^+^ conductances inactivate strongly over this voltage range making their impact weaker than expected (**Fig. S6**). By contrast, the sustained slow K^+^ conductance (delayed rectifier) strongly activates during NMDAR spikes and truncates their duration (**Fig. S6**). Increasing the K^+^ conductances to match levels recently reported experimentally [36], increased the threshold of NMDAR spikes, limited their spread to neighbouring branches and was particularly effective at reducing their decay time (**Fig. S7**). However, the time course of the resulting dendritic spikes generated under these conditions was markedly different from NMDAR spikes recorded experimentally in L5 pyramidal cells [29], [36] and another recent study estimated much lower densities of K^+^ conductances in the tuft region [87] comparable to that in the original L5 model [29]. Nevertheless, slow K^+^ conductances appear well placed to modulate spatio-temporal integration in L5 pyramidal cells. SK type channels in spines, which are activated by local Ca^2+^ influx though NMDARs [88], may also truncate NMDAR spikes (**Fig. S8**). Thus the level of expression of the various dendritic conductances and their precise voltage and Ca^2+^ dependencies are likely to tune the spatio-temporal integration. Irrespective of the natural configuration of the dendritic conductances present *in vivo*, our results using a passive L5 model, show that the spatially distributed NMDAR conductance arising from ongoing network activity will extend spatio-temporal integration unless other dendritic conductances are specifically configured to counteract this basic property. Indeed, spatially distributed glutamate-bound NMDARs arising from ongoing network activity extend the toolbox of dendritic conductances available to pyramidal cells, potentially enabling them to perform a wider range of behaviours.

### Enhanced spatial and temporal integration during background network activity

The fine dendrites of pyramidal cells have traditionally been thought to act as coincidence detectors on fast time scales [89], due to their fast local membrane time constant [31], [90]. However, when depolarized, slowly decaying synaptic NMDAR conductances set the dendritic EPSP waveform, extending the integration window to tens of milliseconds [35]. The voltage dependent properties of NMDARs have also been shown to extend the time window for integration, underlying the supra-linear response of the second of a pair of synaptic inputs onto the basal dendrites of L5 pyramidal cells [32]. Moreover, temporal integration can be extended further when pairs of bursts are used [73], [74]. Our simulations extend these findings by showing that a spatially distributed NMDAR conductance arising from *in vivo*-like background synaptic input can extend both the spatial and temporal integration in L5 pyramidal cells by providing both depolarization [27] and a spatially distributed NMDAR conductance that sustains the NMDAR spike plateau depolarization. Interestingly, these mechanisms act in the opposite direction to passive membrane properties, which reduce the temporal integration window during elevated synaptic input, due to increased membrane shunting by synaptic conductances.

Our simulations show that background synaptic input can lower the number of coincident synaptic inputs required to trigger an NMDAR spike in a distal branch. This suggests that NMDAR spikes are much more likely to occur *in vivo* during network activity than *in vitro* when the network is largely quiescent. Another important consequence of spatially distributed NMDAR conductance arising from background network activity is that it enables active propagation of NMDAR-mediated regenerative events into neighbouring branches, resulting in a multi-branch regenerative plateau potential. This prediction is consistent with recent *in vivo* recordings of NMDAR spikes in L2/3 pyramidal cells, which reported that 83% of events occurred in multiple branches [46]. Spatially distributed NMDAR conductance is therefore well suited for integrating excitatory input that is distributed across the branches of the dendritic tree [39], [40]. Our simulations show that under *in vivo*-like levels of background input our L5 model could integrate spatially and temporally dispersed synaptic input. This suggests that spatial clustering of synaptic inputs required for triggering NMDAR spikes under quiescent *in vitro* conditions [35], [36], [64] may not be necessary *in vivo*. Hence, while spatial clustering of synaptic input onto the dendrites of pyramidal cells has been found [42], [91], our results suggest it is not a prerequisite for activating the tuft of L5 pyramidal cells. Indeed, our finding that network activity extends spatio-temporal integration may explain how NMDAR spikes contribute to orientation selective tuning in L2/3 pyramidal cells in mouse V1 [47], when the synaptic inputs onto these cells are spatially distributed [39].

Widespread NMDAR spikes have been reported in the dendrites of L4 spiny stellate neurons of rat barrel cortex, during whisker deflection [45]. The multiple hot spots of Ca^2+^ influx observed are consistent with a small number of distributed thalamo-cortical inputs being amplified by the dendritic NMDAR conductance arising from local network activity, but alternative scenarios are also possible. In L2/3 pyramidal cells in somatosensory cortex, both spontaneous and hind paw-evoked NMDAR spikes are localized to ∼30 µm regions of the dendritic tree and usually encompass multiple branches. These events are effective at triggering APs but do not appear to involve a dendritic Ca^2+^ spike [46]. More widespread [Ca^2+^] signals have been observed in the tuft of L5 pyramidal cells in awake animals performing active whisker touch, when vibrissal sensory input is combined with L1 input arising from the primary motor cortex [10]. Similar tuft responses could be reproduced *in vitro* when apical trunk Ca^2+^ spikes were paired with dendritic tuft depolarization or synaptic input. This suggests that *in vivo* tuft plateau potentials and Ca^2+^ influx are caused by apical dendritic Ca^2+^ spikes spreading into the tuft when it is depolarized by L1 input. Although, Ca^2+^ conductances are clearly important [10], network activity-dependent distributed NMDAR conductance could also contribute to this widespread Ca^2+^ influx. Indeed, recent *in vivo* recordings from CA1 pyramidal cells show that both NMDARs and Ca^2+^ channels are required to generate the slow, widespread regenerative dendritic events that underlie complex spike bursts [92]. These results suggest that the densities of both glutamate-bound NMDARs and Ca^2+^ channels can set the spatial spread of regenerative dendritic events in pyramidal cells. Our results highlight the role of spatially distributed NMDAR conductances in pyramidal cells under *in vivo* conditions, by showing how they could enable these cells to integrate both spatially clustered and spatially distributed inputs over longer timescales than previously thought.

### Implications for cortical processing

The finding that synaptic input and nonlinear membrane conductances can produce local regenerative dendritic events has lead to the idea that individual dendritic branches can operate as independent thresholding units [33], [73], [93] enabling a pyramidal cell to act as a powerful pattern separator [34]. In this scenario, individual branches could act as feature detectors [77] and neuronal firing could report the coincident occurrence of these features. However, our results showing that NMDAR spikes can spread into neighbouring dendritic branches suggest that they may not operate independently and are more effective at triggering APs than anticipated, consistent with recent experimental findings [46]. This enhancement in spatio-temporal integration during network activity is likely to come at the cost of loss of power to discriminate different spatial patterns of synaptic input. That said, our results do not exclude the possibility that L5 pyramidal cells can still act as coincidence detectors and perform pattern discrimination, since our simulations show that altering the level of dendritic inhibition or the density of voltage-gated channels on the dendrite can tune the spatio-temporal properties of the tuft. Coincidence detection and pattern discrimination could be enhanced by raising the threshold for dendritic spikes, truncating their duration and decoupling dendritic branches. This could be achieved by expressing high levels of K^+^ channels [36], [94], which have been shown to compartmentalize dendritic responses in CA1 pyramidal cells and through experience-dependent adjustment of synaptic weights onto specific branches through NMDAR-dependent Ca^2+^ influx [95]. Moreover, recent work suggests that GIRK channels can also modulate NMDAR spike duration in a branch specific manner [78]. Our results suggest that these slowly modulated dendritic mechanisms are complemented, *in vivo*, by faster dynamic modulation conferred by the background synaptic excitation and inhibition arising from ongoing network activity. Thus, both the past and the present activity state of the network determines which spatio-temporal patterns of synaptic input a L5 pyramidal cell can respond to.

Network activity-dependent spatio-temporal integration could have important implications for cortico-cortical and thalamo-cortical signalling. While global network activity is maintained at a relatively constant level due to tight inhibitory control [16], [18], [71], [85], the activity of sub-networks of excitatory neurons is likely to vary widely, due to common receptive field properties and preferential local synaptic connectivity [96]. If the elevated activity in L2/3 pyramidal cell ensembles observed during sensory input [96] increases synaptic input onto the apical dendrites of subsets of L5 pyramidal cells [4], [97], the resulting increase in NMDAR conductance will enable those L5 pyramidal cells to integrate both spatio-temporally clustered and dispersed signals. This property suggests that the tuft could combine different modalities, such as motor, sensory and emotional state, which span a wide range of timescales. This effect is predicted to be particularly strong when the apical tuft is disinhibited, as has been observed when motor and sensory systems are engaged together [48]. Although speculative, network-dependent enhancement of spatio-temporal integration could also enable L5 pyramidal cells to communicate selectively across sensory and motor cortical areas, in a context dependent manner. Such reconfigurable functional network connectivity has been reviewed in the context of neuronal gain modulation [98] and could be involved in high-level cortical function including attention [76], [99], [100] and learning [9]. Our results strengthen these concepts by providing a new mechanism that links network activity to the spatio-temporal properties of dendritic integration.

## Materials and Methods

For full methodological details see Supplemental Experimental Procedures.

### The L5 pyramidal cell model

The version of the L5 pyramidal cell model [29] available from ModelDB (Accession number: 124043) was used as a starting point for our simulations. Synaptic input was added and all simulations were generated in neuroConstruct (neuroConstruct.org) [51] and run in the NEURON 6.2 simulation environment [101]. The default parameters of the model are listed in **Text S1** and any changes are noted in the figure legends. Simulations were carried out with a fixed integration time step of 0.025 ms and voltages were recorded from the soma and the proximal segment of terminal dendritic branches.

### Synaptic input

The AMPAR conductance (*gAMPA*) component of each quantal glutamatergic synapse was modelled with an instantaneous rise and a single exponential decay:
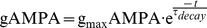
Where the maximal peak conductance (*g_max_* AMPA) was 0.5 nS and the decay time constant (τ_decay_) was 2 ms [56].

The NMDAR conductance (*gNMDA*) component of each quantal glutamatergic synapse was modelled with an exponential rise and decay, and a voltage-dependence function to mimic Mg^2+^ block:
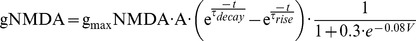
Where the normalization factor A is:
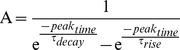

*g_max_* NMDA is the maximal peak unblocked conductance (1 nS). Time constants for the rise (τ_rise_) and decay (τ_decay_) were 3 ms and 70 ms, respectively [76], [97]. The NMDAR model also included Ca^2+^-dependent inactivation (CDI) of the NMDAR current. This was implemented by multiplying the *gNMDA* by *e^−h^*, where the change in *h* at each time step (*h*′) is as follows:
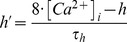
and 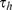 = 1000 ms.

Quantal GABAergic synapses were modelled with an exponential rise and decay:
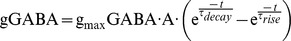
The fast GABA_A_ receptor component had a maximal peak conductance (g_max_GABA) of 0.5 nS, a τ_rise_ = 0.3 ms and a τ_decay_ = 10 ms [66]. *A* is the peak normalization factor as defined above and the Cl^−^ reverse potential was −75 mV. Synapse arising from neurogliaform (NGF) cells, also had a slow GABA_B_ receptor component with a τ_rise_ = 50 ms and a τ_decay_ = 80 ms [70] and an onset delay of 10 ms to account for the activation of the K^+^ channels. The maximal peak conductance (g_max_GABA = 0.06 nS) was estimated from [61] who reported that the GABA_B_ component accounts for ∼1/5 of the peak IPSP for NGF cell to L5 pyramidal cell connections. The conductance was obtained by scaling 0.1 nS by the ratio between the driving forces of the GABA_A_ and GABA_B_ components using a K^+^ reverse potential of −87 mV.

Two groups of synaptic input were defined. Those that mimicked a stimulus-evoked event and those that mimicked asynchronous background network activity. For simulations where specific dendritic branches were stimulated, one of the 28 terminal branches was randomly selected and the stimulus evoked quantal synaptic inputs randomly placed along it. For spatially distributed stimulus-evoked inputs, quantal synaptic inputs were randomly distributed over the entire apical tuft (Blue region, **Fig. 1A**) Each stimulus-evoked input was driven by an independent Poisson train with a frequency that was inversely related to duration of the stimulus, thereby maintaining an average of a single quantal conductance per synapse, independent of stimulus duration. For background excitatory synaptic input, 900–1500 glutamatergic synapses were placed randomly over the apical dendritic region (starting from ∼500 µm from soma). Each of these synapses was driven independently with a Poisson train with a mean rate of 0.85 Hz. Background inhibitory synaptic input was implemented either by 130 GABA_A_ receptor mediated synapses, each driven with a 3 Hz random train (2×SOM configuration) or by a mix of 65 pure GABA_A_ receptor-mediated synapses firing random trains at 3 Hz and 13 GABA_A_/GABA_B_ receptor-mediated connections (10 synapses each) firing random trains at 14 Hz (SOM+NGF configuration). In both cases this gave a time averaged GABA_A_ receptor conductance of 1.5 times the background AMPAR conductance, comparable to that measured experimentally [16], [71].

### Analysis

Voltage traces from the simulations were analysed in IGOR Pro using NeuroMatic software (http://www.neuromatic.thinkrandom.com/). NMDAR spikes were identified with a threshold crossing criteria of −30 mV (**Fig. S1**; unless stated otherwise). Dendritic branch and neuronal I-O functions were fit using a sigmoid function with the form:
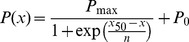
where *n* is the rate, *P_0_* is the offset, *P_max_* is the maximum probability of activation and x*_50_* is the number of synapses or stimulated branches at which P(x) reaches half maximum.

Errors are presented ± standard deviation. Distributions were compared with the Kolmogorov-Smirnov test and considered significant at the p<0.05 level.

## Supporting Information

Figure S1 **NMDAR spike identification criteria.** (**A1–4**) Voltage in randomly selected terminal branches stimulated with 3–30 synapses (blue EPSP, red NMDAR spikes) in the absence of background synaptic input. Voltage responses were classified by eye as EPSPs or NMDAR spikes on basis of shape. (**B1–4**) As for (A), but with 1500 background excitatory synapses. (**C1–4**) As for (A), but with balanced background synaptic input (EXH/INH;2×SOM; I/E = 1.5). (**D**) Relationship between decay time and peak depolarization of single events during control. Due to variability in both parameters (introduced by the random spatiotemporal distribution of the input, variable input resistance and variable branch length) the populations of EPSPs and NMDAR spikes are partly overlapping. During the 15 synapses stimulation a simple threshold criteria at −30 mV (dashed line) misclassified 10% of the NMDAR spikes and 13% of EPSPs (N = 480). (**E**) Relationship between decay time and peak depolarization of single events during background excitation. Distributions of EPSPs and NMDAR spikes are reliably separated by the −30 mV threshold with only 1% NMDAR spikes classified as EPSPs and none of the EPSPs were misclassified. (**F**) Relationship between decay time and peak depolarization of single events during balanced background synaptic input. Distributions of EPSPs and NMDAR spikes are reliably separated by the −30 mV threshold with 4% of the NMDAR spikes classified as EPSPs and 1% of EPSPs classified as NMDAR spikes.
(TIF)

Figure S2 **GABA_A_ and GABA_B_ receptor mediated inhibition.** (**A**) Conductance profile for GABA_A_ receptor synaptic component (red) and GABA_B_ receptor synaptic component mediated by K^+^ conductance (green). (**B**) Somatic IPSP produced by a single mixed GABA_A_/GABA_B_ synapse on a terminal apical branch.
(TIF)

Figure S3 **Voltage depolarization alone cannot account for increased probability and efficacy of NMDAR spikes during excitatory background activity.** (**A1**) Cartoon of L5 pyramidal cell indicating the area where currents were injected (red) in order to produce the average depolarization produced by the background activity of 1500 excitatory synapses in the apical tuft. (**A2**) Probability of NMDAR spike occurrence (P(NMDAR spike)) versus number of stimulated synapses, during control (black dashed line), background excitatory synaptic activity (blue line), apical current injection (red line) and AMPAR-only background synaptic activity (6000 synapses, green) reproducing average depolarization and fluctuations (A3 inset). (**A3**) Probability of action potential P(AP) occurrence versus number of stimulated apical branches (30 synapses per branch) during different conditions. (**B1**) Cartoon indicating location of current injection to produce the average depolarization produced by the background activity of 1500 excitatory synapses in the Ca^2+^ spike initiation zone. (**B2**) P(AP) versus number of stimulated branches, during control (black dashed line) background excitatory synaptic activity (blue line) and Ca^2+^ zone depolarization (red line). (**B3**) Absolute decrease in number of stimulated branches required to trigger an AP (P = 0.5) during apical synaptic input (blue) and Ca^2+^ zone depolarization. (**C1**) Cartoon indicating location of current injection to produce the average depolarization produced by the background activity of 1500 excitatory synapses at the soma. (**C2**) P(AP) versus number of stimulated branches, during control (black dashed line), background excitatory synaptic activity (blue line) and somatic depolarization (red line). (**C3**) Absolute decrease in number of stimulated branches required to trigger an AP (P = 0.5) during apical synaptic input and during somatic depolarization.
(TIF)

Figure S4 **Effect of changing amplitude and time course of the NMDAR synaptic conductance on NMDAR spikes.** (**A**) Average probability of triggering an NMDAR spike (P(NMDA spike)) in a terminal branch versus number of nearly synchronous stimulus-evoked synapses for peak synaptic NMDAR conductances of 1.0 nS (blue) and 0.5 nS (red) with (filled marker, solid lines) and without (empty markers, dashed lines) background activity from 1500 excitatory synapses. Because of the higher AMPA/NMDA ratio, NMDAR spikes were identified by eye from their decay time (>15 ms) rather than peak depolarization for this simulation. (**B**) P(NMDA spike) versus number of nearly synchronous stimulus-evoked synapses for quantal NMDAR conductance amplitudes of 1.0 nS (blue) to 1.5 nS (red) with (filled marker, solid lines) and without (empty markers, dashed lines) background activity of 900 excitatory synapses (reduced because increased *gNMDA* triggered NMDAR spikes with background activity from 1500 excitatory synapses). (**C**) P(NMDA spike) versus number of nearly synchronous stimulus-evoked synapses for NMDAR decay time constants of 70 ms (original, blue) and 35 ms (red) with (filled marker, solid lines) and without (empty markers, dashed lines) background activity from 1500 excitatory synapses. Note: to compensate for the faster decay kinetics the background input rate was doubled (1.7 Hz) in order to deliver the same time averaged NMDAR conductance and depolarization. (**D**) NMDAR spike decay time distributions (N = 100 across randomly selected branches) during control (dashed lines) and during background excitatory activity of 1500 synapses (solid lines), in the original model (70 ms; blue) and in the model with faster NMDAR kinetics (35 ms; red). With a decay constant of 35 ms NMDAR spikes are significantly shorter, both during control and during background excitatory activity. However, the increase over control produced by background was significant (Kolmogorov-Smirnov test, P<0.05).
(TIF)

Figure S5 **Effect of changing dendritic Na^+^, Ca^2+^ and I_h_ conductances on changes in NMDAR spike threshold and duration during background excitatory activity.** (**A1**) Average probability of triggering an NMDAR spike (P(NMDA spike)) versus number of nearly synchronous stimulus-evoked synapses in the original model (black) and in a model where Na^+^ channels were removed from the apical tree (red) measured in the absence (empty markers, dashed lines) and presence of background activity from 1500 excitatory synapses (filled markers, solid lines). (**A2**) NMDAR spike decay time distribution (N = 100 trials across randomly selected branches) for conditions in (A1). (**B1**) Average probability of triggering an NMDAR spike (P(NMDA spike)) versus number of nearly synchronous stimulus-evoked synapses in the original model (black) and in a model where L-type Ca^2+^ channels (Ca-L) were removed from the apical tree including Ca^2+^ initiation zone, during control (black) and background excitatory activity (**B2**) NMDAR spike decay time distribution (N = 100 trials across randomly selected branches) for conditions in (B1). (**C1**) Average probability of triggering an NMDAR spike (P(NMDA spike)) versus number of nearly synchronous stimulus-evoked synapses in the original model (black) and in a model where Ih current was exponentially increased from 0 to 40 pS/µm^2^ along the apical tuft (red) measured in the absence (empty markers, dashed lines) and presence of 1500 background excitatory inputs (filled markers, solid lines). (**C2**) NMDAR spike decay time distribution (N = 100 trials across randomly selected branches) for conditions in (C1).
(TIF)

Figure S6 **Effect of increasing dendritic K^+^ conductances on changes in NMDAR spike threshold and duration during background excitatory activity.** (**A1**) Average probability of triggering an NMDAR spike (P(NMDA spike)) versus number of nearly synchronous stimulus-evoked synapses in the original model (black) and in a model where K^+^ A-type (K_A_) channels density was doubled (60 pS/µm^2^) in the apical tree (red), measured in the absence (empty markers, dashed lines) and presence of 1500 background excitatory input (filled markers, solid lines). (**A2**) NMDAR spike decay time distribution (N = 100 trials across randomly selected branches) for conditions in (A1). (**B1**) Average probability of triggering an NMDAR spike (P(NMDA spike)) versus number of nearly synchronous stimulus-evoked synapses in the original model (black) and in a model where K^+^ delayed rectifier (K_dr_) channels density was increased ten-fold (10 pS/µm^2^) in the apical tree (red), during control (empty markers, dashed lines) and background excitatory activity (filled markers, solid lines). (**B2**) NMDAR spike decay time distribution (N = 100 trials across randomly selected branches) for conditions in (B1).
(TIF)

Figure S7 **Effect of increasing K^+^ conductances on the dendritic tuft to match levels reported by Harnett et al. 2013.** (**A**) Average probability of triggering an NMDAR spike (P(NMDA spike)) versus number of nearly synchronous stimulus-evoked synapses in the original model (black) and in a model where apical K_A_ density was increased to 77 pS/µm^2^ and apical K_dr_ density to 23 pS/µm^2^ to match estimates from [36] (blue), during control (empty markers, dashed lines) and background excitatory activity (filled markers, solid lines). (**B**) NMDAR spike decay time distribution (N = 100 trials across randomly selected branches) for conditions in (A). The decay time was computed as the time when the voltage decayed to 30% of the peak depolarization (instead of 37%) to account for the fluctuations present when the K^+^ conductance was increased (see G). (**C**) Average profile of NMDA spikes (N = 100) triggered in the original model (grey) and in a model with K^+^ conductances equal to 1 and 2 times the levels reported by [36] (blue and red respectively). Twice as much K^+^ conductance was required to completely counteract the background NMDAR component, matching the average NMDA spike recorded during background excitation with 6000 AMPAR-only synapse, which matched the depolarization during mixed AMPAR+NMDAR excitatory background (green). Note: the time course of this dendritic spike is markedly different from NMDA spikes recorded experimentally in L5 pyramidal cells [29] (**D**) Average number (N = 28) of additional branches activated during background input ± SE when a single branch is nearly synchronously stimulated in the modified model (blue) compared to the original model (grey). (**E**) Example voltage traces from branch 12 (red) and branch 9 (blue) in the absence (dashed lines) and presence of 1500 background excitatory inputs (solid lines). (**F**) Peak depolarization induced by an NMDAR spike in branch 12 versus terminal branch number in absence (empty marks, dotted line) and presence (filled marks, solid line) of background activity. (**G**) Local voltage profile of an NMDA spike (dark blue, upper panel) triggered in the modified model by the near synchronous activation of 30 glutamatergic synapses. Current generated by each of the 30 NMDAR synapses (light blue, middle panel) compared with the current densities of the three major ions (Na^+^ in green, K^+^ in red and, Ca^2+^ in yellow). Note: another recent study [87] estimated a much lower density of K_dr_ (3 pS/µm^2^) in the apical dendrites of L5 pyramidal cells.
(TIF)

Figure S8 **Effect of Ca^2+^-dependent local feedback mechanisms on NMDAR spikes and spatio-temporal integration.** (**A**) Individual quantal synaptic NMDAR conductance with Ca^2+^-dependent inactivation (CDI) present in the original model (black trace) and when CDI was removed (red line), both recorded at −60 mV. (**B**) Ca^2+^ current through the NMDAR quantal conductance with (black trace) and without (red line) CDI at −60 mV, together with scaling factor for NMDA conductance (lower plot). (**C**) Branch voltage during a single NMDAR spike with (dashed traces) and without (solid traces) CDI of NMDARs, in the absence (red) and presence of background activity from 1500 excitatory synapses (blue). The effect of CDI became evident during the longer plateau potential, because Ca^2+^ accumulation lead to shortening of NMDAR conductances. (**D**) NMDAR spike decay time distribution (N = 100 trials across randomly selected branches) for conditions in (C). During background excitatory input the average NMDAR spike duration increased 4.8 fold with background excitation without CDI, compared to 1.4 in the original model. (**E**) Average NMDAR spike decay time in the absence (control, open markers) and presence of background excitatory input from 1500 excitatory synapses (solid marker), with different levels of negative feedback implemented with an SK-like Ca^2+^-dependent K^+^ conductance at each synapse (SK_syn_). SK_syn_ at a density of 40 pS/synapse reproduced the average decay time in the original model (orange symbol). (**F**) Cumulative distributions of NMDAR spike decay times with different levels of SK_syn_ (light blue 0 pS, dark blue 40 pS, red 120 pS) in the absence (control, dashed lines) and presence of background excitation (solid markers). All levels tested show a significant increase during background excitation compared to control (Kolmogorov-Smirnov test: P<0.05). (**G**) Average probability of action potential (P(AP)) (N = 20 trials) with different levels of negative feedback (light blue 0 pS, dark blue 40 pS, red 120 pS) in the absence (control, open markers, dashed lines) and presence of background excitatory input (filled symbols, solid lines), for different numbers of distributed synapses stimulated with random trains at 10 Hz for 100 ms.
(TIF)

Movie S1 **Movie 1 shows the membrane voltage in the Layer 5 Pyramidal cell model during nearly synchronous stimulus evoked synaptic stimulation with 30 synapses on a terminal dendritic branch (each synapse firing at 200 Hz from 100–105 ms).** The voltage response to the synaptic input is shown under 3 conditions: 1) Control with no background activity. 2) With background activity from 1500 excitatory synapses firing randomly at 0.85 Hz. 3) With balanced background synaptic input including inhibition from SOM-like (GABA_A_) and NGF-like (GABA_A_ and GABA_B_) mediated inhibition. While the spread of depolarisation in the control case is limited to the stimulated branch and its near neighbours, the excitatory background input causes regenerative depolarisation in branches further away. The spread of depolarisation is also widespread for balanced background synaptic input, but the presence of inhibition shortens the duration compared with background excitation alone.
(AVI)

Movie S2 **Movie 2 shows the membrane voltage in the Layer 5 Pyramidal cell model during nearly synchronous stimulus evoked synaptic stimulation of 100 synapses spatially distributed over the apical tuft (10 Hz from 100–200 ms) under 3 conditions: 1) Control with no background activity.** 2) With background activity from 1500 excitatory synapses firing randomly at 0.85 Hz3) With balanced background synaptic input including inhibition from SOM-like (GABA_A_) and NGF-like (GABA_A_ and GABA_B_) mediated inhibition. Only in the case of stimulation in the presence of excitatory input did the spatially distributed synaptic input trigger somatic action potentials which back propagates into the apical tree.
(AVI)

Text S1 **Supporting information.** Supplemental methods, results, table and references.
(DOCX)
