# Supplementary figures and images for: Glutamate-Bound NMDARs Arising from In Vivo-like Network Activity Extend Spatio-temporal Integration in a L5 Cortical Pyramidal Cell Model
Source: PLoS Comput Biol. 2014 Apr 24;10(4):e1003590. doi: 10.1371/journal.pcbi.1003590 (PMC3998913; doi:10.1371/journal.pcbi.1003590)

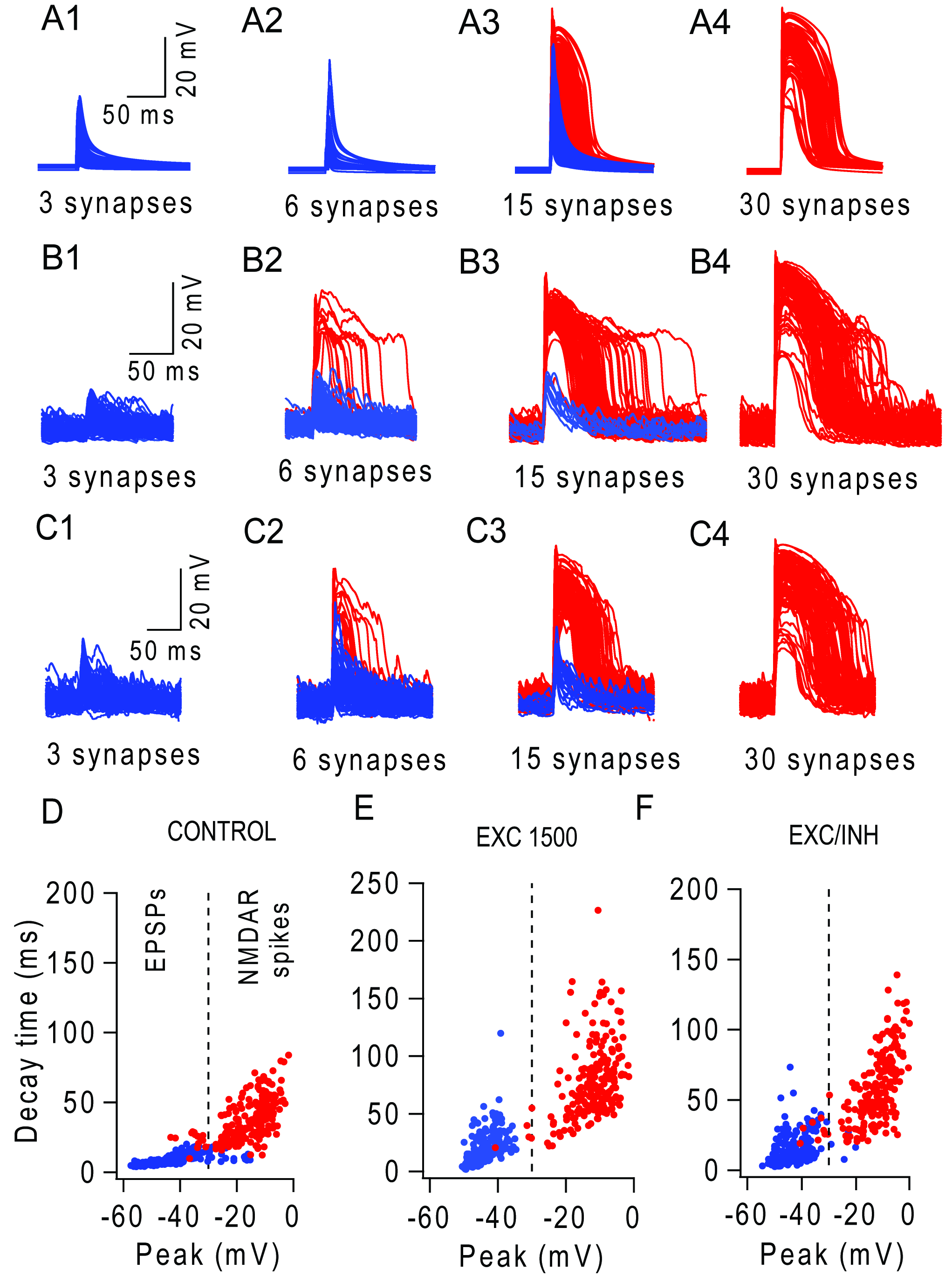

Supplement: Figure S1 — NMDAR spike identification criteria. (A1–4) Voltage in randomly selected terminal branches stimulated with 3–30 synapses (blue EPSP, red NMDAR spikes) in the absence of background synaptic input. Voltage responses were classified by eye as EPSPs or NMDAR spikes on basis of shape. (B1–4) As for (A), but with 1500 background excitatory synapses. (C1–4) As for (A), but with balanced background synaptic input (EXH/INH;2×SOM; I/E = 1.5). (D) Relationship between decay time and peak depolarization of single events during control. Due to variability in both parameters (introduced by the random spatiotemporal distribution of the input, variable input resistance and variable branch length) the populations of EPSPs and NMDAR spikes are partly overlapping. During the 15 synapses stimulation a simple threshold criteria at −30 mV (dashed line) misclassified 10% of the NMDAR spikes and 13% of EPSPs (N = 480). (E) Relationship between decay time and peak depolarization of single events during background excitation. Distributions of EPSPs and NMDAR spikes are reliably separated by the −30 mV threshold with only 1% NMDAR spikes classified as EPSPs and none of the EPSPs were misclassified. (F) Relationship between decay time and peak depolarization of single events during balanced background synaptic input. Distributions of EPSPs and NMDAR spikes are reliably separated by the −30 mV threshold with 4% of the NMDAR spikes classified as EPSPs and 1% of EPSPs classified as NMDAR spikes. (TIF) [file pcbi.1003590.s001.tif]

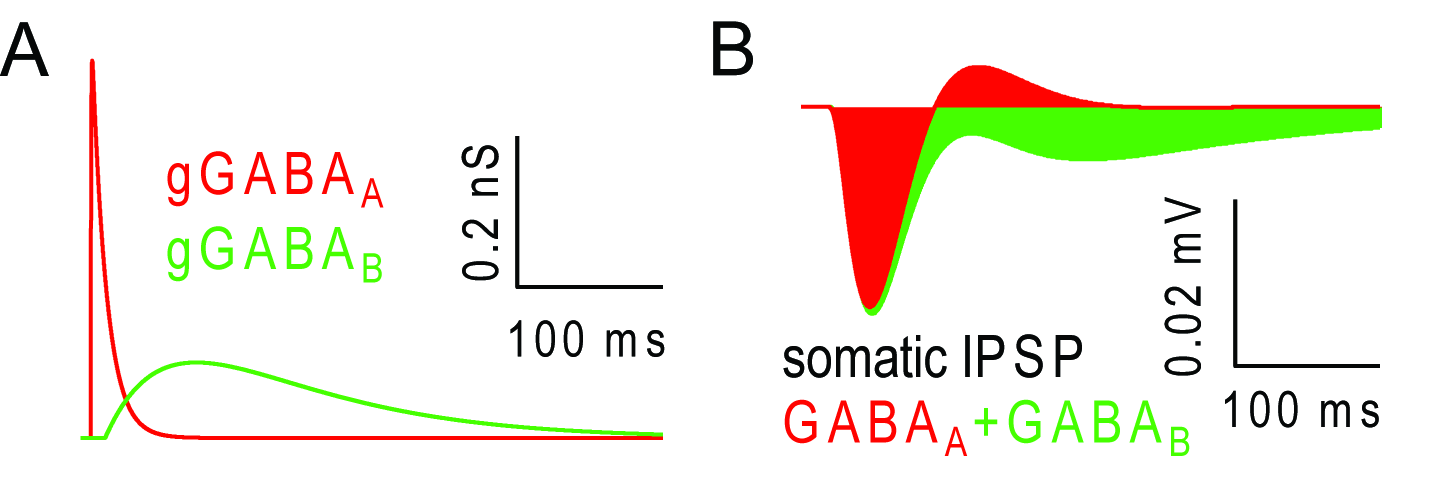

Supplement: Figure S2 — GABAA and GABAB receptor mediated inhibition. (A) Conductance profile for GABAA receptor synaptic component (red) and GABAB receptor synaptic component mediated by K+ conductance (green). (B) Somatic IPSP produced by a single mixed GABAA/GABAB synapse on a terminal apical branch. (TIF) [file pcbi.1003590.s002.tif]

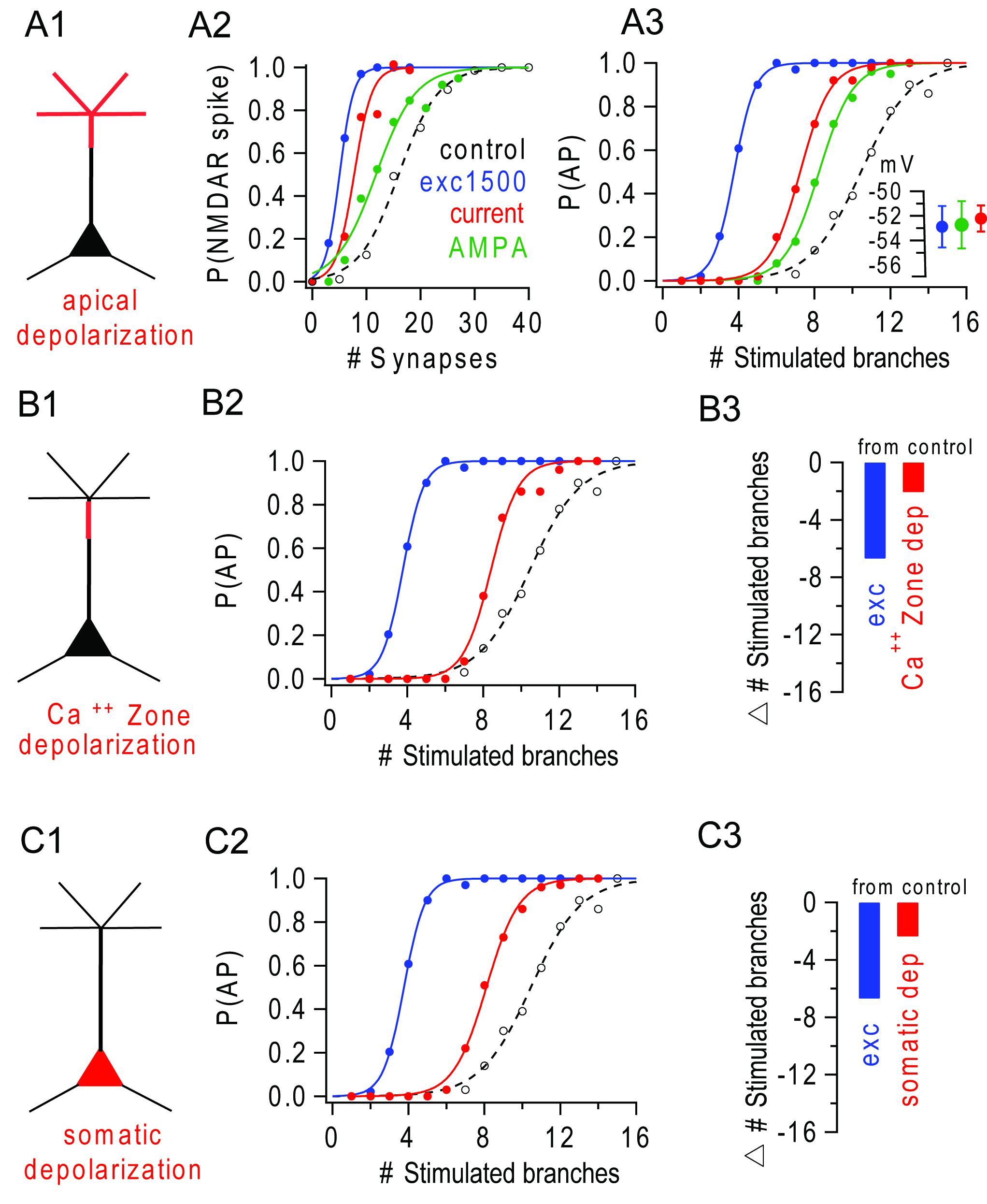

Supplement: Figure S3 — Voltage depolarization alone cannot account for increased probability and efficacy of NMDAR spikes during excitatory background activity. (A1) Cartoon of L5 pyramidal cell indicating the area where currents were injected (red) in order to produce the average depolarization produced by the background activity of 1500 excitatory synapses in the apical tuft. (A2) Probability of NMDAR spike occurrence (P(NMDAR spike)) versus number of stimulated synapses, during control (black dashed line), background excitatory synaptic activity (blue line), apical current injection (red line) and AMPAR-only background synaptic activity (6000 synapses, green) reproducing average depolarization and fluctuations (A3 inset). (A3) Probability of action potential P(AP) occurrence versus number of stimulated apical branches (30 synapses per branch) during different conditions. (B1) Cartoon indicating location of current injection to produce the average depolarization produced by the background activity of 1500 excitatory synapses in the Ca2+ spike initiation zone. (B2) P(AP) versus number of stimulated branches, during control (black dashed line) background excitatory synaptic activity (blue line) and Ca2+ zone depolarization (red line). (B3) Absolute decrease in number of stimulated branches required to trigger an AP (P = 0.5) during apical synaptic input (blue) and Ca2+ zone depolarization. (C1) Cartoon indicating location of current injection to produce the average depolarization produced by the background activity of 1500 excitatory synapses at the soma. (C2) P(AP) versus number of stimulated branches, during control (black dashed line), background excitatory synaptic activity (blue line) and somatic depolarization (red line). (C3) Absolute decrease in number of stimulated branches required to trigger an AP (P = 0.5) during apical synaptic input and during somatic depolarization. (TIF) [file pcbi.1003590.s003.tif]

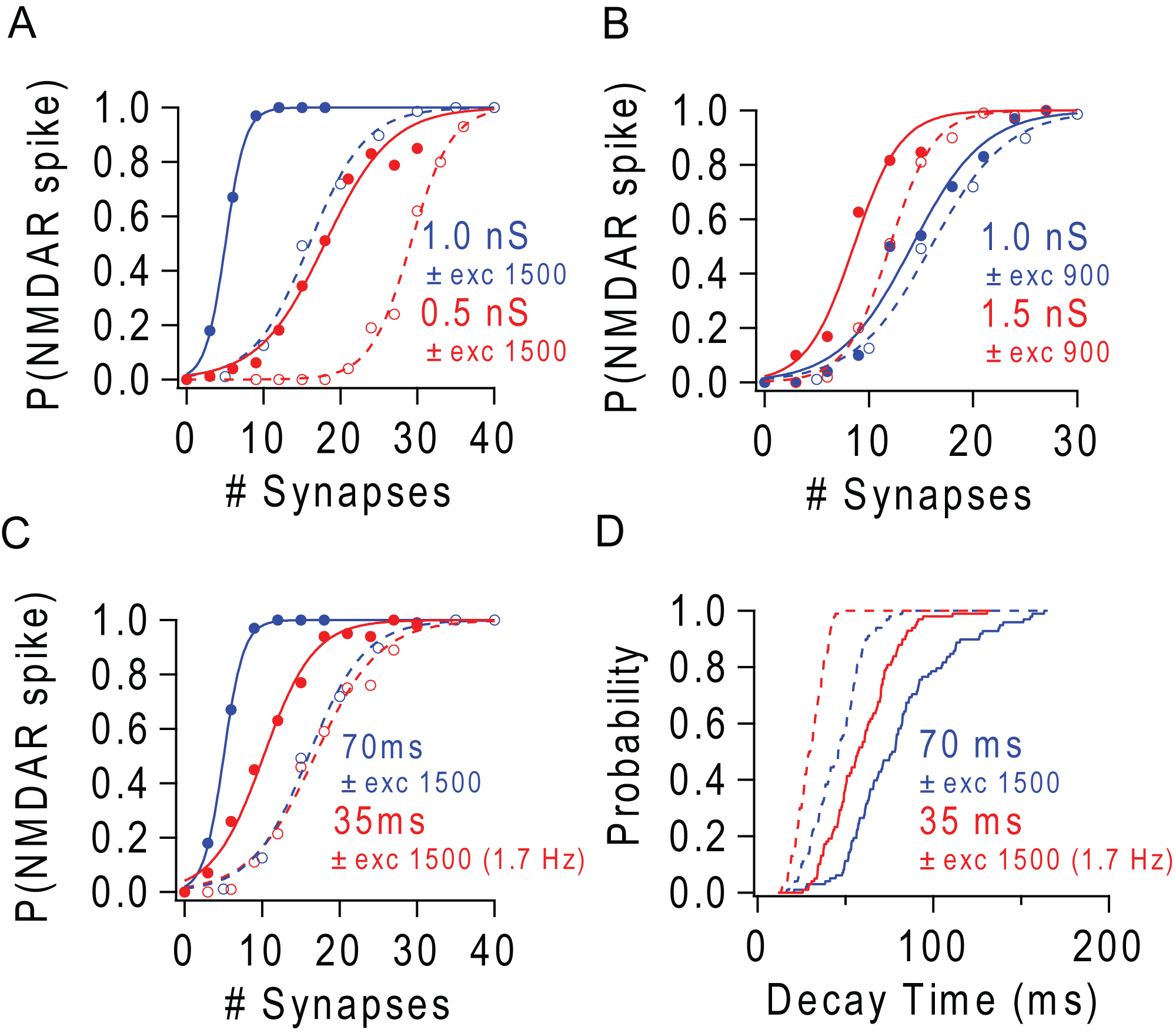

Supplement: Figure S4 — Effect of changing amplitude and time course of the NMDAR synaptic conductance on NMDAR spikes. (A) Average probability of triggering an NMDAR spike (P(NMDA spike)) in a terminal branch versus number of nearly synchronous stimulus-evoked synapses for peak synaptic NMDAR conductances of 1.0 nS (blue) and 0.5 nS (red) with (filled marker, solid lines) and without (empty markers, dashed lines) background activity from 1500 excitatory synapses. Because of the higher AMPA/NMDA ratio, NMDAR spikes were identified by eye from their decay time (>15 ms) rather than peak depolarization for this simulation. (B) P(NMDA spike) versus number of nearly synchronous stimulus-evoked synapses for quantal NMDAR conductance amplitudes of 1.0 nS (blue) to 1.5 nS (red) with (filled marker, solid lines) and without (empty markers, dashed lines) background activity of 900 excitatory synapses (reduced because increased gNMDA triggered NMDAR spikes with background activity from 1500 excitatory synapses). (C) P(NMDA spike) versus number of nearly synchronous stimulus-evoked synapses for NMDAR decay time constants of 70 ms (original, blue) and 35 ms (red) with (filled marker, solid lines) and without (empty markers, dashed lines) background activity from 1500 excitatory synapses. Note: to compensate for the faster decay kinetics the background input rate was doubled (1.7 Hz) in order to deliver the same time averaged NMDAR conductance and depolarization. (D) NMDAR spike decay time distributions (N = 100 across randomly selected branches) during control (dashed lines) and during background excitatory activity of 1500 synapses (solid lines), in the original model (70 ms; blue) and in the model with faster NMDAR kinetics (35 ms; red). With a decay constant of 35 ms NMDAR spikes are significantly shorter, both during control and during background excitatory activity. However, the increase over control produced by background was significant (Kolmogorov-Smirnov test, P<0.05). (TIF) [file pcbi.1003590.s004.tif]

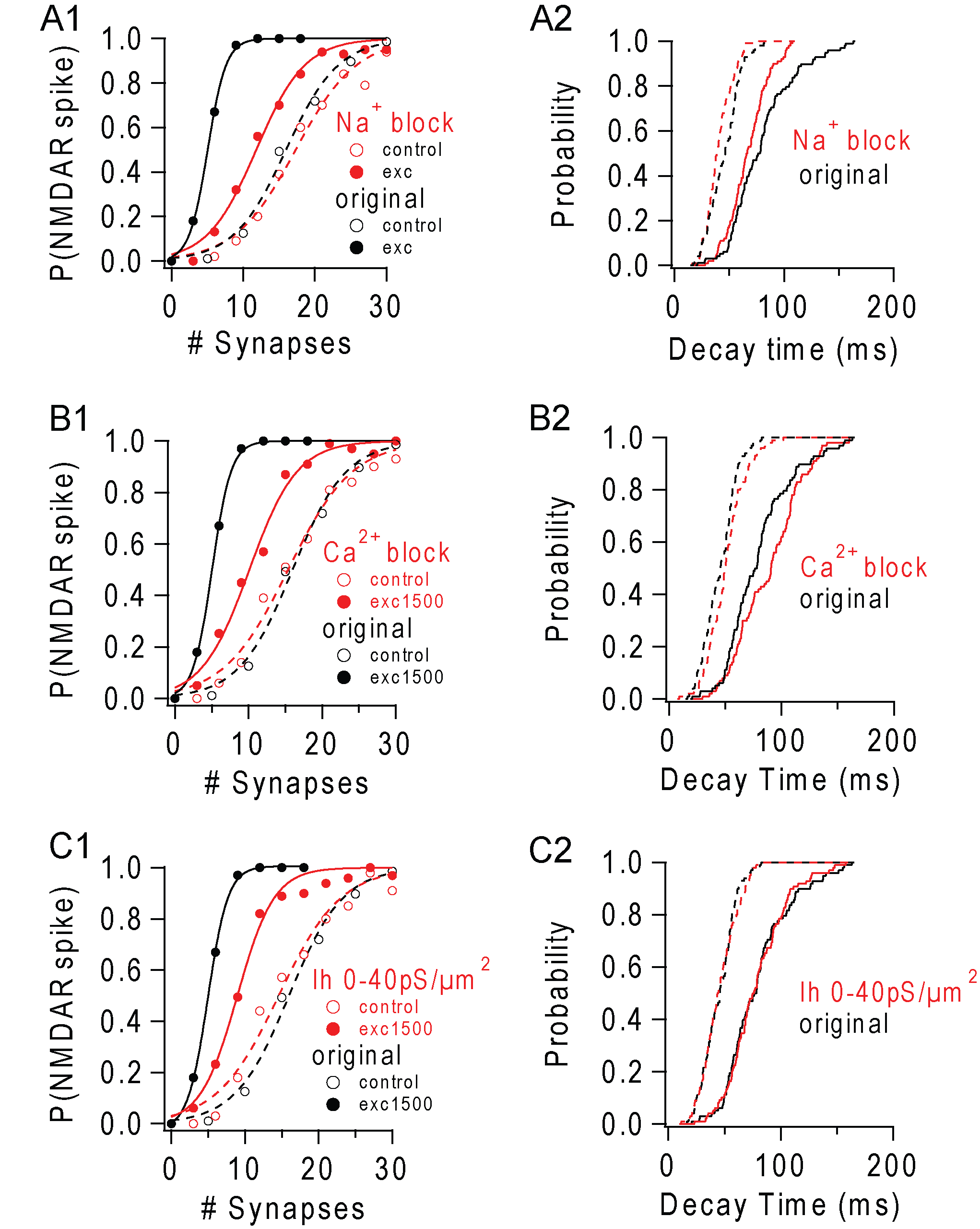

Supplement: Figure S5 — Effect of changing dendritic Na+, Ca2+ and Ih conductances on changes in NMDAR spike threshold and duration during background excitatory activity. (A1) Average probability of triggering an NMDAR spike (P(NMDA spike)) versus number of nearly synchronous stimulus-evoked synapses in the original model (black) and in a model where Na+ channels were removed from the apical tree (red) measured in the absence (empty markers, dashed lines) and presence of background activity from 1500 excitatory synapses (filled markers, solid lines). (A2) NMDAR spike decay time distribution (N = 100 trials across randomly selected branches) for conditions in (A1). (B1) Average probability of triggering an NMDAR spike (P(NMDA spike)) versus number of nearly synchronous stimulus-evoked synapses in the original model (black) and in a model where L-type Ca2+ channels (Ca-L) were removed from the apical tree including Ca2+ initiation zone, during control (black) and background excitatory activity (B2) NMDAR spike decay time distribution (N = 100 trials across randomly selected branches) for conditions in (B1). (C1) Average probability of triggering an NMDAR spike (P(NMDA spike)) versus number of nearly synchronous stimulus-evoked synapses in the original model (black) and in a model where Ih current was exponentially increased from 0 to 40 pS/µm2 along the apical tuft (red) measured in the absence (empty markers, dashed lines) and presence of 1500 background excitatory inputs (filled markers, solid lines). (C2) NMDAR spike decay time distribution (N = 100 trials across randomly selected branches) for conditions in (C1). (TIF) [file pcbi.1003590.s005.tif]

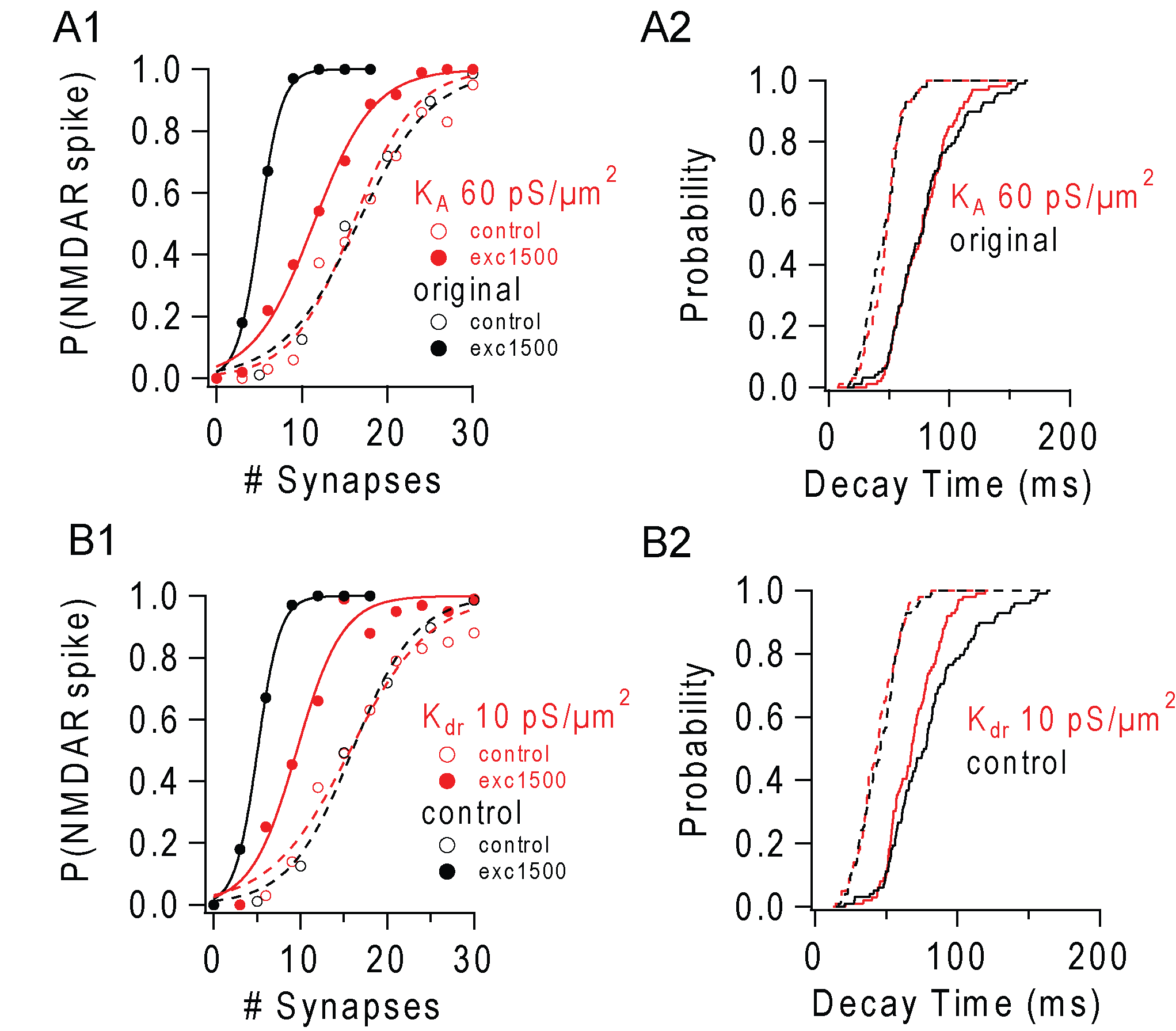

Supplement: Figure S6 — Effect of increasing dendritic K+ conductances on changes in NMDAR spike threshold and duration during background excitatory activity. (A1) Average probability of triggering an NMDAR spike (P(NMDA spike)) versus number of nearly synchronous stimulus-evoked synapses in the original model (black) and in a model where K+ A-type (KA) channels density was doubled (60 pS/µm2) in the apical tree (red), measured in the absence (empty markers, dashed lines) and presence of 1500 background excitatory input (filled markers, solid lines). (A2) NMDAR spike decay time distribution (N = 100 trials across randomly selected branches) for conditions in (A1). (B1) Average probability of triggering an NMDAR spike (P(NMDA spike)) versus number of nearly synchronous stimulus-evoked synapses in the original model (black) and in a model where K+ delayed rectifier (Kdr) channels density was increased ten-fold (10 pS/µm2) in the apical tree (red), during control (empty markers, dashed lines) and background excitatory activity (filled markers, solid lines). (B2) NMDAR spike decay time distribution (N = 100 trials across randomly selected branches) for conditions in (B1). (TIF) [file pcbi.1003590.s006.tif]

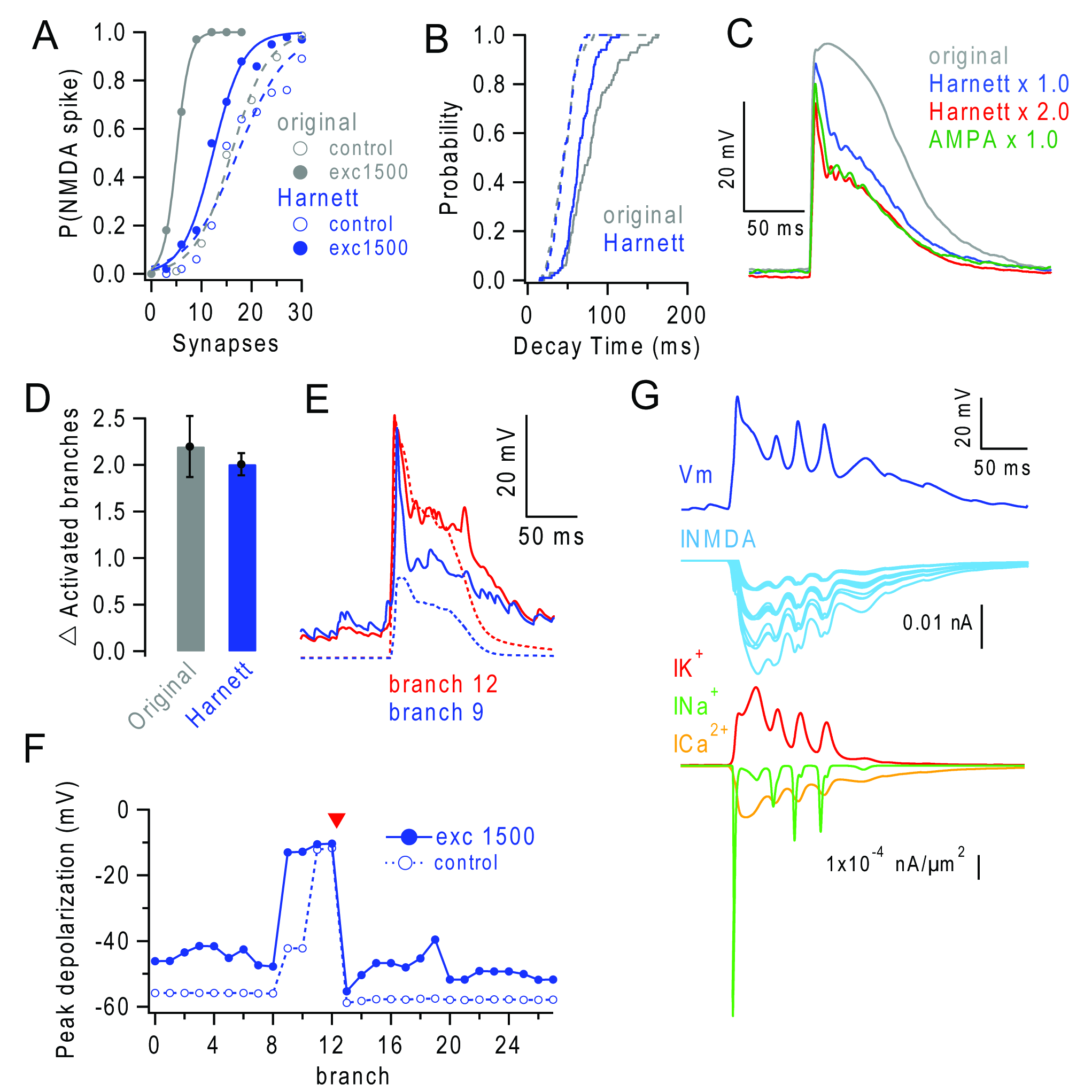

Supplement: Figure S7 — Effect of increasing K+ conductances on the dendritic tuft to match levels reported by Harnett et al. 2013. (A) Average probability of triggering an NMDAR spike (P(NMDA spike)) versus number of nearly synchronous stimulus-evoked synapses in the original model (black) and in a model where apical KA density was increased to 77 pS/µm2 and apical Kdr density to 23 pS/µm2 to match estimates from [36] (blue), during control (empty markers, dashed lines) and background excitatory activity (filled markers, solid lines). (B) NMDAR spike decay time distribution (N = 100 trials across randomly selected branches) for conditions in (A). The decay time was computed as the time when the voltage decayed to 30% of the peak depolarization (instead of 37%) to account for the fluctuations present when the K+ conductance was increased (see G). (C) Average profile of NMDA spikes (N = 100) triggered in the original model (grey) and in a model with K+ conductances equal to 1 and 2 times the levels reported by [36] (blue and red respectively). Twice as much K+ conductance was required to completely counteract the background NMDAR component, matching the average NMDA spike recorded during background excitation with 6000 AMPAR-only synapse, which matched the depolarization during mixed AMPAR+NMDAR excitatory background (green). Note: the time course of this dendritic spike is markedly different from NMDA spikes recorded experimentally in L5 pyramidal cells [29] (D) Average number (N = 28) of additional branches activated during background input ± SE when a single branch is nearly synchronously stimulated in the modified model (blue) compared to the original model (grey). (E) Example voltage traces from branch 12 (red) and branch 9 (blue) in the absence (dashed lines) and presence of 1500 background excitatory inputs (solid lines). (F) Peak depolarization induced by an NMDAR spike in branch 12 versus terminal branch number in absence (empty marks, dotted line) and presence (filled marks, solid [file pcbi.1003590.s007.tif]

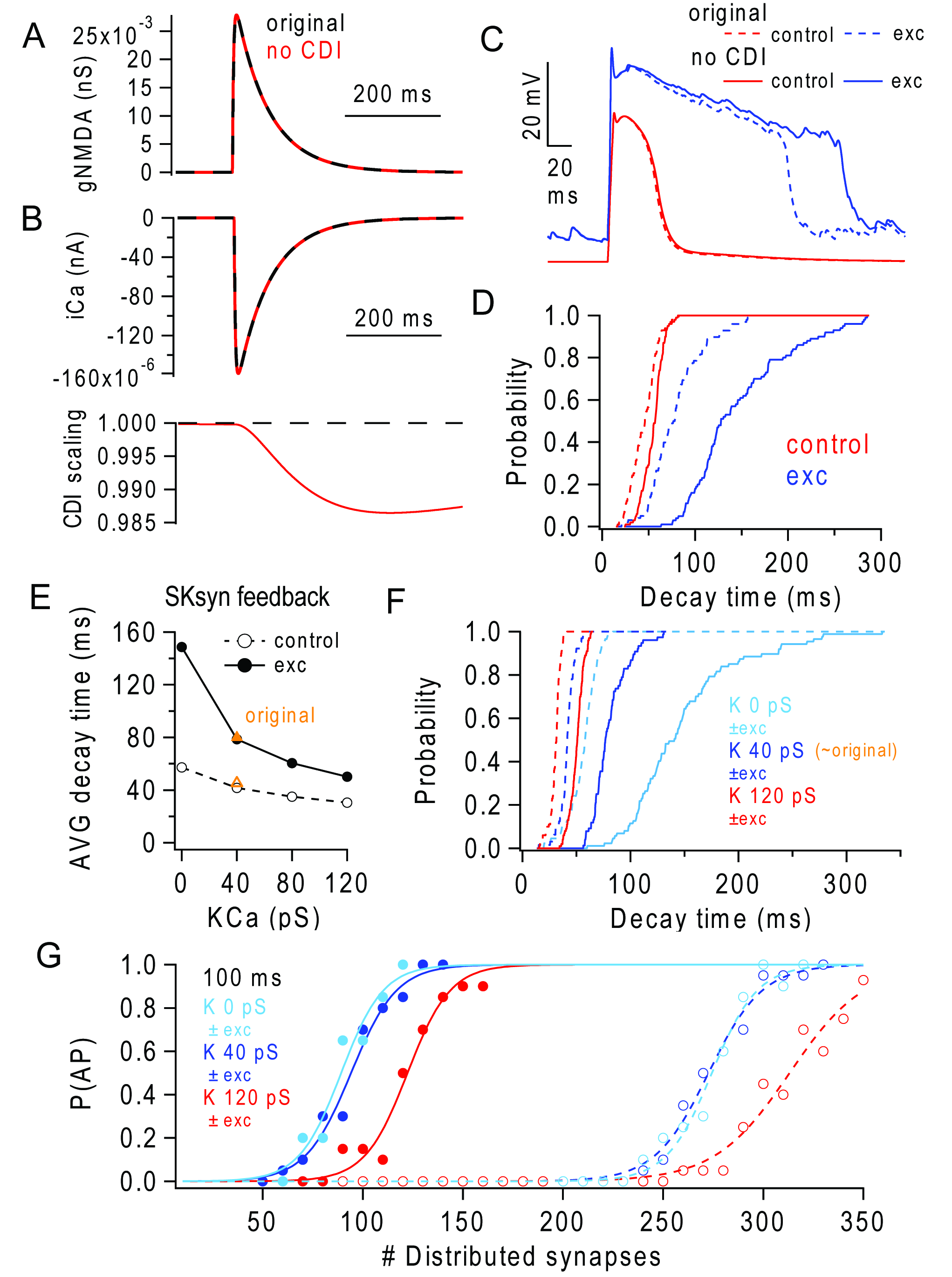

Supplement: Figure S8 — Effect of Ca2+-dependent local feedback mechanisms on NMDAR spikes and spatio-temporal integration. (A) Individual quantal synaptic NMDAR conductance with Ca2+-dependent inactivation (CDI) present in the original model (black trace) and when CDI was removed (red line), both recorded at −60 mV. (B) Ca2+ current through the NMDAR quantal conductance with (black trace) and without (red line) CDI at −60 mV, together with scaling factor for NMDA conductance (lower plot). (C) Branch voltage during a single NMDAR spike with (dashed traces) and without (solid traces) CDI of NMDARs, in the absence (red) and presence of background activity from 1500 excitatory synapses (blue). The effect of CDI became evident during the longer plateau potential, because Ca2+ accumulation lead to shortening of NMDAR conductances. (D) NMDAR spike decay time distribution (N = 100 trials across randomly selected branches) for conditions in (C). During background excitatory input the average NMDAR spike duration increased 4.8 fold with background excitation without CDI, compared to 1.4 in the original model. (E) Average NMDAR spike decay time in the absence (control, open markers) and presence of background excitatory input from 1500 excitatory synapses (solid marker), with different levels of negative feedback implemented with an SK-like Ca2+-dependent K+ conductance at each synapse (SKsyn). SKsyn at a density of 40 pS/synapse reproduced the average decay time in the original model (orange symbol). (F) Cumulative distributions of NMDAR spike decay times with different levels of SKsyn (light blue 0 pS, dark blue 40 pS, red 120 pS) in the absence (control, dashed lines) and presence of background excitation (solid markers). All levels tested show a significant increase during background excitation compared to control (Kolmogorov-Smirnov test: P<0.05). (G) Average probability of action potential (P(AP)) (N = 20 trials) with different levels of negative feedback (light blue 0 pS, dark blue 40 pS, red [file pcbi.1003590.s008.tif]
