# Supplementary material for: Glutamate-Bound NMDARs Arising from In Vivo-like Network Activity Extend Spatio-temporal Integration in a L5 Cortical Pyramidal Cell Model
Source: PLoS Comput Biol. 2014 Apr 24;10(4):e1003590. doi: 10.1371/journal.pcbi.1003590 (PMC3998913; doi:10.1371/journal.pcbi.1003590)
Supplement: Text S1 — Supporting information. Supplemental methods, results, table and references. (DOCX) [file pcbi.1003590.s011.docx]

Supplemental Information

## Glutamate-bound NMDARs arising from *in vivo*-like network activity extend spatio-temporal integration in L5 cortical pyramidal cell model

Matteo Farinella, Daniel T Ruedt, Padraig Gleeson, Frederic Lanore and

R. Angus Silver

**Inventory**

**1. Supplemental Methods**……………………………………………….......………......2

**2. Supplemental Results** ……………………………………………….......…...…......7

**3. Supplemental Table** S1…………………………………………………..…..............10

**4. Supplemental References**…………………………………………………..…..........11

**1 Supplemental Methods**

**Compartmental model of L5 pyramidal cell.**

The version of the L5 pyramidal neuron model [1] available from ModelDB ([Senselab.med.yale.edu/ModelDB/](http://senselab.med.yale.edu/ModelDB/) Accession number: 124043) was used as a starting point for our simulations. This was translated into the NeuroML description language (NeuroML.org) [2], making it simulator independent (the scripts for these simulations can be obtained from the Open Source Brain repository: [http://www.opensourcebrain.org/projects/farinellaetal_nmdaspikes](http://www.opensourcebrain.org/projects/farinellaetal_nmdaspikes)). All the simulations were generated in neuroConstruct (neuroConstruct.org) [3] and run in the NEURON 6.2 simulation environment [4]. The morphology was based on a L5 tufted pyramidal neuron from a young adult Wistar rat (36 ± 5 day old; [1]). The multi-compartmental model included 189 sections, each divided in 11 NEURON segments, of total length 11,927 μm. Spine morphologies were not explicitly modelled [1]. Electrical properties including the nine membrane conductances together with their distributions are detailed in **Table S1**. When cell and synapse parameters were varied for control simulations their values are mentioned in the figure legend. For the passive model all conductances were removed from the cell except for the passive leak conductance.

**Synaptic conductances**

The AMPAR conductance (*gAMPA*) component of each quantal glutamatergic synapse was modelled with an instantaneous rise and a single exponential decay:

$$\text{gAMPA=g}_{\max}\text{AMPA}\cdot\text{e}^{\frac{-t}{\tau_{decay}}}$$

Where the maximal peak conductance (*g*_max_*AMPA*) was 0.5 nS and the decay time constant (τ_decay_) was 2 ms [5].

The NMDAR conductance (*gNMDA*) component of each quantal glutamatergic synapse was modelled with an exponential rise and decay, and a voltage-dependence function to mimic Mg^2+^ block:

$$\text{gNMDA=g}_{\max}\text{NMDA}\cdot\text{A}\cdot\left(\text{e}^{\frac{-t}{\tau_{decay}}}-\text{e}^{\frac{-t}{\tau_{rise}}}\right)\cdot\frac{1}{1+0.3\cdot e^{-0.08V}}$$

Where the normalization factor A is:

$$\text{A=}\frac{1}{\text{e}^{\frac{-peak_{time}}{\tau_{decay}}}-\text{e}^{\frac{-peak_{time}}{\tau_{rise}}}}$$

And the time to reach the max conductance is:

$$peak_{time}=\log\left(\frac{\tau_{decay}}{\tau_{rise}}\right)\cdot\frac{\tau_{rise}\cdot\tau_{decay}}{\tau_{decay}-\tau_{rise}}$$

The maximal peak unblocked conductance (*g_max_ NMDA*) was 1 nS. This gave an NMDAR/AMPAR peak conductance ratio of 2, which is within the range reported for pyramidal cells [1,6–9]. Moreover, the time averaged conductance ratio is equivalent to the one used in [1]. The time constants for the *gNMDA* rise (τ_rise_) and decay (τ_decay_) were 3 ms and 70 ms, respectively. This decay time course is in the range reported for cortico-cortical projections (50 ms) and long range cortico-cortical connections (100 ms) [10,11] which are likely to convey the majority of the apical input simulated. The value used also matches the GluN2B containing NMDARs in adult animals (~100 ms) [12]. For simulations where faster NMDAR kinetics were examined τ_decay_ was reduced to 35 ms (**Fig. S4**). The voltage dependence of the Mg^2+^ block, which mimicked the strong voltage dependence of GluN2A/B containing NMDARs, had a half maximal block of approximately -20 mV [13]. The NMDAR model also included Ca^2+^-dependent inactivation (CDI) of the NMDA current. This was implemented by multiplying the *gNMDA* by *e^-h^*, where the change in *h* at each time step (*h*’) is as follows:

$$h'\text{=}\frac{8\cdot\left[Ca^{2+}\right]_{i}-h}{\tau_{h}}$$

and τ_h_= 1000 ms.

GABA receptor mediated synaptic conductances arising from all interneuron types were modelled with an exponential rise and decay:

$$\text{gGABA=g}_{\max}\text{GABA}\cdot\text{A}\cdot\left(\text{e}^{\frac{-t}{\tau_{decay}}}-\text{e}^{\frac{-t}{\tau_{rise}}}\right)$$

The fast GABA_A_ receptor component had a maximal peak conductance (g_max_GABA) of 0.5 nS, a τ_rise_ = 0.3 ms and a τ_decay_ = 10 ms [14]. *A* is the peak normalization factor as defined above and the GABA_A_R Cl^-^ reverse potential was -75 mV. Synapse arising from neurogliaform (NGF) cells, also had a slow GABA_B_ receptor component with a τ_rise_ = 50 ms and a τ_decay_ = 80 ms [15] and an onset delay of 10 ms to account for the activation of the K^+^ channels. The maximal peak conductance (g_max_GABA= 0.06 nS) was estimated from [16] who reported that the GABA_B_ receptor component accounts for ~1/5 of the peak IPSP for NGF cell to L5 pyramidal cell connections. The conductance was obtained by scaling 0.1 nS by the ratio between the driving forces of the GABA_A_R and GABA_B_R components using a K^+^ reversal potential of -87 mV.

**Distribution of synapses on the dendritic tree and their firing rates**

Excitatory synaptic input onto specific branches was implemented with neuroConstruct by randomly selecting one of the 28 terminal branches and then randomly distributing the required number of quantal synaptic inputs along it. Each synaptic input was driven by an independent Poisson train set to a frequency that resulted in approximately one quantal conductance per synapse, irrespective of the duration of the stimulus. For example, a 200 Hz train was used for the 5 ms window and a 20 Hz input was applied for the 50 ms stimuli. For simulations that included background excitatory synaptic input, 900-1500 glutamatergic synapses were placed randomly over the apical dendritic region starting at 500 µm from soma (except where explicitly stated). Each of these background synapses was driven independently with a different Poisson train with a mean rate of 0.85 Hz.

To simulate the inhibitory synaptic input under *in vivo*-like conditions as closely as possible, fast GABA_A_ receptor mediated synaptic input from somatostatin (SOM) expressing interneurons and mixed fast and slow GABA_A_/GABA_B_ receptor mediated synaptic input arising from NGF cells were implemented. Inhibition from SOM cells consisted of 65 fast GABA_A_-type synapses (i.e. 13 cells with 5 contacts) distributed randomly over the apical dendritic region each driven at 3 Hz (as recorded from awake mice *in vivo* [17]) while NGF input consisted of 13 cells firing random trains at 14 Hz (as recorded from awake mice *in vivo* [17]) that had both a GABA_A_ and a GABA_B_ components and 10 anatomical contacts randomly distributed over the apical tuft [18]. These numbers were determined by assuming that there approximately the same number of SOM and NGF interneurons innervating the apical dendrites of a tufted L5 cell and that their combined time averaged GABA_A_ receptor-mediated conductance was 1.5 times the total background AMPAR conductance as measured experimentally [19,20]. To examine the contribution of the slow GABA_B_ receptor-mediated inhibition we carried out some simulations were we omitted NGF cells and doubled the number of GABA_A_ receptor synapses arising from SOM cells (2xSOM condition 130 synapses).

**Calcium-mediated negative feedback mechanisms and compartmentalization.**

The original model [1] included two mechanisms that provided negative feedback during strong synaptic activation: NMDAR Ca^2+^-dependent inactivation (CDI) [21–23] and Ca^2+^ activated potassium channels. These were both driven by a Ca^2+^ pool that is filled via Ca^2+^ influx through NMDARs and L-type Ca^2+^ channels. While the contribution of these mechanisms is not well defined in L5 pyramidal neurons, both of these mechanisms provided local negative feedback counteracting and truncating NMDAR spikes. They are activated during NMDAR spikes and their presence is required to match the model NMDAR spike duration to experimental dendritic recordings [1]. However, implementation of such mechanisms is complicated in NEURON, because internal Ca^2+^ is not able to diffuse between neighbouring compartments. During non-uniform synaptic distributions, compartments with higher synaptic density may therefore reach a higher [Ca^2+^] leading to stronger recruitment of Ca^2+^-dependent K^+^ channels (K_Ca_) and a strong Ca^2+^-dependent inactivation of NMDA receptors, preferentially curtailing NMDAR spikes in these segments. However, in neighbouring compartments which receive fewer synapses, the Ca^2+^ concentration may never rise to a level sufficient to activate K_Ca_ channels. Also, with this configuration CDI of the NMDAR could be affected by Ca^2+^ influx from other synapses (but which seems unlikely given the presence of spines). To control for these potential problems we carried out a series of control simulations. These ranged from removing all the negative feedback mechanisms (**Fig. S8**), to implementing a simplified SK-like potassium conductance point process mechanism that was driven only by the Ca^2+^ current through NMDARs at each synapse, and was therefore independent of the size of dendritic compartments. While the absolute duration of the NMDAR spike was affected by these manipulations, our key results showing background activity modulates spatio-temporal integration was unaffected (**Fig. S8**).

**Simulation configurations and properties of driving synaptic input**

Simulations were carried out with a fixed integration step of 0.025 ms, although model behaviour was tested for convergence with shorter values of d*t* (data not shown). Synapses were stimulated 300-500 ms after the onset of the background input to allow the membrane potential to reach a quasi steady-state value. Voltages were recorded at the soma and from the more proximal segment of each stimulated apical dendrite. The probability of triggering an NMDAR spike during control was estimated by stimulating each of 28 terminal branches with 5, 10, 15, 20, 25, 30 and 40 synchronous synapses (i.e. 5 ms 200 Hz burst). During background activity the resolution was increased to 3, 6, 9, 12, 15, 18, 21, 24, 27, 30 and 40 synapses to account for a lower threshold and steeper response. When studying the temporal integration properties, branches or distributed synaptic inputs were triggered at random times within 50, 100, 150, 200 ms windows. When multiple branches were stimulated each of the randomly selected branches was stimulated with a cluster of 30 synapses firing random trains at 200 Hz for a 5 ms window. This ensured a high probability of triggering an NMDA spike in that branch (see **Fig. 1H**). Thus, in **Fig. 3** the “input” refers to the time frame during which the 5 ms stimulations of the selected branches were randomly delivered. To study spread of regenerative events into neighbouring branches, single branches were triggered in randomly selected terminal branches, while recording from all the 28 branches. The peak depolarization in each branch was measured with and without background activity. Additional regenerative events were identified when the difference between the peak depolarization during background and the peak depolarization during control exceed 13 mV. When the model was stimulated with spatially distributed input (**Fig. 4 and 5D**), 20-400 synapses were randomly placed on the entire apical tuft (as for the background input) and driven with 200 Hz trains for 5 ms, 20 Hz trains for 50 ms, 10 Hz trains for 100 ms or 5 Hz for 200 ms. Thus, in **Fig. 4** and the other figures where spatially distributed input is shown, “input” represents the stimulation duration of random trains of synaptic input. The original model assigned to each section 11 segments in NEURON, regardless of their length. To ensure our results were not affected by this approximation we measured NMDAR spike decay distributions in a recompartmentalized model produced by neuroConstruct, where each anatomical section is divided into a number of segments proportional to the electrotonic length, ensuring that no segment is longer than 0.01 of the local space constant. In the resulting recompartmentalized model some NMDAR spikes were shorter and some longer, but on average the results were similar to the original model (data not shown).

**Analysis**

Voltage traces were sampled with neuroConstruct from NEURON simulations and analysed in IGOR Pro (Wavemetrics) using NeuroMatic software (<http://www.neuromatic.thinkrandom.com/>). NMDAR spikes were identified with a threshold crossing criteria of - 30 mV (unless stated otherwise). This estimated the number of NMDAR spikes over a range of conditions (**Fig. S1**). For putative NMDAR spikes the following parameters were then measured:

- *Baseline voltage* (mV): the average membrane potential recorded in the dendritic branch before the triggering stimulus.
- *Peak voltage* (mV): the maximum membrane potential recorded in the dendritic branch.
- *Amplitude* (mV): difference between the peak depolarization and the baseline depolarization.
- *Decay time* (ms): computed as the time when the voltage decayed to 37% of the peak depolarization, unless otherwise stated.

Dendritic branch and neuronal input-output functions were fit using a sigmoid equation of the form:

$$P(x)\text{=}\frac{P_{\max}}{1+\exp\left(\frac{x_{50}-x}{n}\right)}+P_{0}$$

where *n* is the rate, *P_max_* is the maximum probability, *P_0_* is the base probability (typically 1 and 0, respectively) and *X*_50_ is the number of synapses or branches at which *P*(*x*) reaches half maximum. The effect of background activity on the I-O relationship was estimated from the change in half-maximal response from the control case.

Action potentials (APs) were identified with a 0 mV somatic voltage threshold. The model produced both single APs and short stereotypical bursts of 3 APs (>60 Hz) depending on the stimulation protocol. Bursts invariably occurred when the model was driven by apical input, due to Ca^2+^ spikes, which are often triggered in the Ca^2+^ hot zone by back propagating APs, similarly to that reported *in vitro* [1]. The probability of APs P(AP) refers to the probability of occurrence of one or more spikes. Errors are presented ± standard deviation (SD). Distributions were compared with the Kolmogorov-Smirnov test and considered significant at the p<0.05 level.

**2 Supplemental results**

**Potential impact of negative feedback mechanisms within spines and NMDAR kinetics**

Local negative feedback mechanisms present in dendritic spines could ‘inhibit’ NMDAR spikes and thus counteract background input dependent elongation of the NMDAR spike. Dendritic spines were not explicitly modelled but an increased dendrite capacitance was implemented in the model [1]. Moreover, some local negative feedback mechanisms which may be active in spines were included in our synaptic models, as mentioned above. The NMDAR model we used included calcium-dependent inactivation (CDI) [21–23], which when removed, produced a larger increase in decay times, from an average of 55 ms during control to 148 ms during background excitatory synaptic input (**Fig. S8A-D**).

An SK-type Ca^2+^-activated potassium channel conductance, shown to be present in spines [24], could also counteract prolonged plateau potentials induced by synaptic input. To explore the potential impact of these channels we implemented an SK-like conductance mechanism at each synapse (SK_syn_) and systematically increased the conductance from 0 to 120 pS/synapse. As expected, the duration of NMDAR spikes became shorter as the SK_syn_ conductance was increased (**Fig. S8E, F**), but even at 120 pS/synapse, which is 3-fold higher than the SK_syn_ conductance density required to match experimentally measured NMDAR spike length during synaptic activation, background excitatory synaptic input substantially increased the NMDAR decay time over control (**Fig. S8F**). Moreover, a comparable shift in the neuronal I-O relationship was observed for spatially and temporally distributed synaptic input over the whole SK_syn_ conductance range explored when background excitatory synaptic input was added (**Fig. S8G**).

We also examined the effect of altering the conductance and kinetics of the synaptic NMDARs. Reducing or increasing the NMDAR conductance altered the branch I-O relationship but did not prevent the basic effect of background excitatory synaptic input on the branch I-O relationship (**Fig. S4A, B**). Speeding up NMDAR kinetics reduced the absolute length of NMDAR spikes both during control and during background excitatory synaptic input; however background input, frequency-scaled to give the same time averaged NMDAR conductance, still produced a significant increase in NMDAR spike duration over control (**Fig. S4C, D**)**.** These results suggest that background excitatory synaptic input-dependent increase in NMDAR spike duration, and the consequent increase in spatial and temporal integration properties of the cell, are robust to negative feedback mechanisms present in dendrites and spines [24,25] and a range of NMDAR conductance and decay kinetics.

**Potential interactions with other voltage-dependent conductances on the dendritic tree.**

Several nonlinear conductances have been characterized in L5 pyramidal cell dendrites [26–29]. In order to examine how these dendritic conductances might facilitate or counteract elongation of NMDAR spikes during background excitatory synaptic input, we explored the effect of varying their density or eliminating them from the apical dendritic region.

Sodium conductances present in the dendrite are expected to facilitate regenerative activity while calcium channels in the tuft are thought to underlie the widespread [Ca^2+^] transients observed *in vivo* [30]. Calcium channels can also mediate Ca^2+^ spikes during strong tuft stimulation or during back propagating APs [31], thereby facilitating the transfer of tuft signals to the Na^+^ spike initiation zone in the axon initial segment [32]. Removal of the Na^+^ conductance from the tuft slowed the rise of NMDAR spikes and increased their threshold in the L5 model as expected (**Fig. S5A1**). It also decreased the average NMDAR spike decay time, although the decay times were still significantly longer during background activity than during control (**Fig. S5A2,** Kolmogorov-Smirnov test p < 0.05). Removal of the Ca^2+^ conductance from the tuft and hot zone reduced tuft excitability, resulting in a higher threshold for NMDAR spikes (**Fig. S5B1**). However, a reduction in threshold and enhanced spike duration over control were observed during background excitatory input (**Fig. S5B1-B2**).

When Ih was increased and distributed non-uniformly from a value of 0 in the most proximal segment of the tuft up to a value of 40 pS/µm^2^ in the terminal branches [27], the resting membrane potential was depolarized by about 3 mV. The elevated membrane potential slightly reduced the effect of background synaptic input on NMDAR spike threshold (**Fig. S5C1**) but had little effect on the NMDAR spike duration, which remained significantly longer during background than during control (**Fig. S5C2,** Kolmogorov-Smirnov test p < 0.05).

High levels of expression of potassium conductances in the tuft are expected to curtail the NMDAR spike-mediated depolarization. Doubling the density of A-type potassium channels in the tuft (60 pS/µm^2^) increased the threshold and reduced the duration of the NMDAR spike depolarization (**Fig. S6A1**), but did not abolish the increased duration with background excitatory synaptic input (**Fig. S6A2**). Increasing the delayed rectifier potassium channel conductance in the tuft from 1 pS/µm^2^ to 10 pS/µm^2^ resulted in shorter NMDAR spikes (**Fig. S6B**). Increasing both conductances (K_A_ = 77 pS/µm^2^, K_dr_ = 23 pS/µm^2^) to match values recently recorded from the tuft of L5 pyramidal neurons for transient and persistent potassium currents [28], had a comparable effect (**Fig. S7**). However, the NMDAR spikes have a different shape to those observed experimentally [1], and even exhibit oscillations, suggesting the model is unstable when the dendritc K^+^ conductance is elevated to these levels. It is interesting to note that another recent study [29] that combined dendritic recordings and pharmacological block to constrain the conductance density of ion channels on the apical dendrite concluded that K^+^ conductances are expressed at a lower density (3 pS/µm^2^), consistent with the Larkum model (1 pS/µm^2^; [1]). Nevertheless, these simulations show that NMDAR spike duration can be modulated by background excitatory input even when the delayed rectifier K^+^ conductance is substantial.

**Note on robustness and limitations of our model-based predictions**

The electrical behaviour of the model, which was generated by the 9 membrane conductances (**Table S1**), reproduced a wide range of experimentally measured dendritic and somatic behaviours [1]. Moreover, the properties of the synaptic conductances, the density of synaptic innervation and background firing rates were all set to experimentally measured values [8,17,33–35]. The results reflect the average behaviour of many instances of the model, where the synaptic input pattern over the dendritic tree is randomly generated and the activity patterns of each synaptic input consists of an independent random train, it therefore takes into account random variation in input statistics and the difference in the properties of individual dendritic branches.

Although we have constrained many of the parameters in the model to experimentally determined values, some mechanisms present in real L5 cells may still be poorly constrained, over simplified, or missing entirely. The control simulations described in the preceding sections tested both the robustness of the mechanisms we have discovered and controlled for potential artefacts arising from the way Ca^2+^ accumulation is handled in the model. While we show several model parameters can alter the excitability and the set point of temporal integration window of the L5 model, it proved difficult to eliminate our basic result showing that background input alters dendritic integration, by altering parameters in the model. Our modelling results therefore show how background network activity could control spatio-temporal integration in a cortical pyramidal cell and the physical plausibility of the underlying NMDAR-mediated mechanisms, but we cannot of course rule out the possibility that mechanisms are present in real cells that are precisely tuned to counteract the robust properties that we observe over a wide range of model conditions.

**3 Supplemental Table**

| **Model parameters** | **global** | **axon** | **soma** | **trunk** | **CaZone** | **Apical** | | **Basal** |
| --- | --- | --- | --- | --- | --- | --- | --- | --- |
| R_i_ (ohmcm) | 85 |  |  |  |  |  |  | |
| C (μFcm^-2^) |  | 1 | 1 | 1 | 1.4 | 1.4 | 1 | |
| E_pas_ (mV) | -70 |  |  |  |  |  |  | |
| E_K_ (mV) | -87 |  |  |  |  |  |  | |
| E_Na_ (mV)  E_Ih_ (mV)  E_Ca_ (mV) | 50  -34  132 |  |  |  |  |  |  | |
| pas (pS/μm^2^) |  | 0.5 | 0.5 | 0.5 | 0.5 | 0.5 | 0.5 | |
| Na |  | 30000 | 50 | 50 | 100 | 40 | 40 | |
| K |  | 8000 | 600 | 1000 |  |  |  | |
| K_dr_ |  | 10000 | 3000 | 600 | 1 | 1 | 1 | |
| K_m_ |  |  | 10 |  |  |  |  | |
| K_A_ |  |  | 30 | 600 | 30 | 30 | 30 | |
| K_Ca_ |  |  | 10 | 1 | 30 | 10 | 10 | |
| Ca_L_ |  |  | 1 | 1 | 10 | 1 | 1 | |
| I_h_ |  |  |  |  | 10 | 10 |  | |
| gNMDA (nS) |  |  |  |  | 1 | 1 |  | |
| rise (ms) |  |  |  |  | 3 | 3 |  | |
| decay (ms)  E_NMDA_ (mV) |  |  |  |  | 70  0 | 70  0 |  | |
| gAMPA (nS) |  |  |  |  | 0.5 | 0.5 |  | |
| rise (ms) |  |  |  |  | 0 | 0 |  | |
| decay (ms)  E_AMPA_ (mV)  gGABA_A_ (nS)  rise (ms)  decay (ms)  E_GABAA_ (mV)  gGABA_B_ (nS)  rise (ms)  decay (ms)  delay (ms)  E_GABAB_ (mV) |  |  |  |  | 2  0  0.5  0.3  10  -75  0.06  50  80  10  -87 | 2  0  0.5  0.3  10  -75  0.06  50  80  10  -87 |  | |

**Table S1. Main parameters of the L5 pyramidal cell model.**

**4 Supplemental References**

1. Larkum M, Nevian T, Sandler M, Polsky A, Schiller J (2009) Synaptic integration in tuft dendrites of layer 5 pyramidal neurons: a new unifying principle. Science 325: 756–760. doi:10.1126/science.1171958.

2. Gleeson P, Crook S, Cannon RC, Hines ML, Billings GO, et al. (2010) NeuroML: a language for describing data driven models of neurons and networks with a high degree of biological detail. PLoS Comput Biol 6: e1000815. doi:10.1371/journal.pcbi.1000815.

3. Gleeson P, Steuber V, Silver RA (2007) neuroConstruct: a tool for modeling networks of neurons in 3D space. Neuron 54: 219–235. doi:10.1016/j.neuron.2007.03.025.

4. Carnevale NT, Hines ML (2009) The NEURON Book. 1st ed. Cambridge University Press.

5. Branco T, Häusser M (2011) Synaptic integration gradients in single cortical pyramidal cell dendrites. Neuron 69: 885–892. doi:10.1016/j.neuron.2011.02.006.

6. Branco T, Clark BA, Häusser M (2010) Dendritic discrimination of temporal input sequences in cortical neurons. Science 329: 1671–1675. doi:10.1126/science.1189664.

7. Kumar SS, Huguenard JR (2003) Pathway-specific differences in subunit composition of synaptic NMDA receptors on pyramidal neurons in neocortex. J Neurosci Off J Soc Neurosci 23: 10074–10083.

8. Markram H, Lübke J, Frotscher M, Roth A, Sakmann B (1997) Physiology and anatomy of synaptic connections between thick tufted pyramidal neurones in the developing rat neocortex. J Physiol 500 ( Pt 2): 409–440.

9. Myme CIO, Sugino K, Turrigiano GG, Nelson SB (2003) The NMDA-to-AMPA Ratio at Synapses Onto Layer 2/3 Pyramidal Neurons Is Conserved Across Prefrontal and Visual Cortices. J Neurophysiol 90: 771 –779. doi:10.1152/jn.00070.2003.

10. Wang H, Stradtman GG 3rd, Wang X-J, Gao W-J (2008) A specialized NMDA receptor function in layer 5 recurrent microcircuitry of the adult rat prefrontal cortex. Proc Natl Acad Sci U S A 105: 16791–16796. doi:10.1073/pnas.0804318105.

11. Williams SR, Atkinson SE (2007) Pathway-specific use-dependent dynamics of excitatory synaptic transmission in rat intracortical circuits. J Physiol 585: 759–777. doi:10.1113/jphysiol.2007.138453.

12. Cull-Candy S, Brickley S, Farrant M (2001) NMDA receptor subunits: diversity, development and disease. Curr Opin Neurobiol 11: 327–335.

13. Kuner T, Schoepfer R (1996) Multiple Structural Elements Determine Subunit Specificity of Mg2+ Block in NMDA Receptor Channels. J Neurosci 16: 3549–3558.

14. Gupta A, Wang Y, Markram H (2000) Organizing principles for a diversity of GABAergic interneurons and synapses in the neocortex. Science 287: 273–278.

15. Sanders H, Berends M, Major G, Goldman MS, Lisman JE (2013) NMDA and GABAB (KIR) Conductances: The “Perfect Couple” for Bistability. J Neurosci 33: 424–429. doi:10.1523/JNEUROSCI.1854-12.2013.

16. Jiang X, Wang G, Lee AJ, Stornetta RL, Zhu JJ (2013) The organization of two new cortical interneuronal circuits. Nat Neurosci 16: 210–218. doi:10.1038/nn.3305.

17. Gentet LJ, Kremer Y, Taniguchi H, Huang ZJ, Staiger JF, et al. (2012) Unique functional properties of somatostatin-expressing GABAergic neurons in mouse barrel cortex. Nat Neurosci 15: 607–612. doi:10.1038/nn.3051.

18. Tamás G, Lőrincz A, Simon A, Szabadics J (2003) Identified Sources and Targets of Slow Inhibition in the Neocortex. Science 299: 1902–1905. doi:10.1126/science.1082053.

19. Haider B, Häusser M, Carandini M (2013) Inhibition dominates sensory responses in the awake cortex. Nature 493: 97–100. doi:10.1038/nature11665.

20. Liu G (2004) Local structural balance and functional interaction of excitatory and inhibitory synapses in hippocampal dendrites. Nat Neurosci 7: 373–379. doi:10.1038/nn1206.

21. Vissel B, Krupp JJ, Heinemann SF, Westbrook GL (2002) Intracellular domains of NR2 alter calcium-dependent inactivation of N-methyl-D-aspartate receptors. Mol Pharmacol 61: 595–605.

22. Kyrozis A, Albuquerque C, Gu J, MacDermott AB (1996) Ca(2+)-dependent inactivation of NMDA receptors: fast kinetics and high Ca2+ sensitivity in rat dorsal horn neurons. J Physiol 495 ( Pt 2): 449–463.

23. Legendre P, Rosenmund C, Westbrook GL (1993) Inactivation of NMDA channels in cultured hippocampal neurons by intracellular calcium. J Neurosci Off J Soc Neurosci 13: 674–684.

24. Ngo-Anh TJ, Bloodgood BL, Lin M, Sabatini BL, Maylie J, et al. (2005) SK channels and NMDA receptors form a Ca2+-mediated feedback loop in dendritic spines. Nat Neurosci 8: 642–649. doi:10.1038/nn1449.

25. Cai X, Liang CW, Muralidharan S, Muralidharan S, Kao JPY, et al. (2004) Unique roles of SK and Kv4.2 potassium channels in dendritic integration. Neuron 44: 351–364. doi:10.1016/j.neuron.2004.09.026.

26. Bekkers JM (2000) Distribution and activation of voltage-gated potassium channels in cell-attached and outside-out patches from large layer 5 cortical pyramidal neurons of the rat. J Physiol 525 Pt 3: 611–620.

27. Berger T, Larkum ME, Lüscher HR (2001) High I(h) channel density in the distal apical dendrite of layer V pyramidal cells increases bidirectional attenuation of EPSPs. J Neurophysiol 85: 855–868.

28. Harnett MT, Xu N-L, Magee JC, Williams SR (2013) Potassium Channels Control the Interaction between Active Dendritic Integration Compartments in Layer 5 Cortical Pyramidal Neurons. Neuron 79: 516–529. doi:10.1016/j.neuron.2013.06.005.

29. Almog M, Korngreen A (2014) A quantitative description of dendritic conductances and its application to dendritic excitation in layer 5 pyramidal neurons. J Neurosci Off J Soc Neurosci 34: 182–196. doi:10.1523/JNEUROSCI.2896-13.2014.

30. Xu N-L, Harnett MT, Williams SR, Huber D, O’Connor DH, et al. (2012) Nonlinear dendritic integration of sensory and motor input during an active sensing task. Nature. doi:10.1038/nature11601.

31. Larkum ME, Zhu JJ, Sakmann B (1999) A new cellular mechanism for coupling inputs arriving at different cortical layers. Nature 398: 338–341. doi:10.1038/18686.

32. Stuart G, Schiller J, Sakmann B (1997) Action potential initiation and propagation in rat neocortical pyramidal neurons. J Physiol 505 ( Pt 3): 617–632.

33. Braitenberg V, Schüz A (1991) Anatomy of the Cortex. Springer-Verlag.

34. Margrie TW, Brecht M, Sakmann B (2002) In vivo, low-resistance, whole-cell recordings from neurons in the anaesthetized and awake mammalian brain. Pflüg Arch Eur J Physiol 444: 491–498. doi:10.1007/s00424-002-0831-z.

35. De Kock CPJ, Bruno RM, Spors H, Sakmann B (2007) Layer- and cell-type-specific suprathreshold stimulus representation in rat primary somatosensory cortex. J Physiol 581: 139–154. doi:10.1113/jphysiol.2006.124321.
